# Supplementary material for: Qualitative study exploring the feasibility of using medication monitors and a differentiated care approach to support adherence among people receiving TB treatment in South Africa
Source: BMJ Open. 2023 Mar 3;13(3):e065202. doi: 10.1136/bmjopen-2022-065202 (PMC9990642; doi:10.1136/bmjopen-2022-065202)
Supplement: Supplementary data [file bmjopen-2022-065202supp001.pdf]

APPENDIX I

STUDY NUMBER: -□□□-□□□□□-□ **AUR2-1-244**

INTERVIEW GUIDE:

DEPARTMENT OF HEALTH KEY STAKEHOLDERS/ FACILITY-BASED STAFF/STUDY STAFF

TB MATE

Introduction

Staff to say out loud before starting interview

- Thank you for agreeing to take part in our research
- We are interested in knowing about your perceptions, thoughts and attitude toward the differentiated model of care, especially with regard to implementation of the medication device. There are no right or wrong answers.
- You do not have to answer any questions if you don't want to. Just say pass and I will move to the next question.
- Your answers to these questions may help us improve TB treatment services.
- All of your answers will be kept confidential. Confidential means we will not tell anyone your answers outside the research group.

Mukora R, *et al. BMJ Open* 2023; 13:e065202. doi: 10.1136/bmjopen-2022-065202

BMJ Publishing Group Limited (BMJ) disclaims all liability and responsibility arising from any reliance placed on this supplemental material which has been supplied by the author(s)

APPENDIX I

STUDY NUMBER: -□□□-□□□□□-□ **AUR2-1-244**

INTERVIEW GUIDE:

DEPARTMENT OF HEALTH KEY STAKEHOLDERS/ FACILITY-BASED STAFF/STUDY STAFF

Introduction and Ground Rules

1. Obtain written informed consent first, before any data are collected.

2. Interviewer to introduce self. Thank you for taking the time to meet with us today. Our names are [*insert names*]___________and we would like to talk to you about the feasibility, acceptability and the quality to which the differentiated care model was implemented. We are doing this project to understand your role, thoughts, attitude and perceptions of the differentiated model of care and particularly to see whether the medical device technology could be integrated and sustained within the TB programme. We want you to be as open and honest when answering. There are no right or wrong answers in this discussion. Please feel free to tell us what you think.

    - Interviewer to explain the ground rules and terms of confidentiality for the interview.
    - The participant does not have to answer any question they do not want to.
    - The information you share will be handled in confidence. (in secret)
    - When we report back on the information collected in this discussion, your comments will not be able to be linked to you specifically.
    - We ask that you also agree not to share anything discussed in this room with others.

3. The interview should take about 1 hour

4. Interviewer to inform the interviewee that the in-depth interview will be digitally recorded to make sure that all themes are captured. Turn the audio-recorder on and ask for verbal permission again to digitally record the session. While the audio-recorder is running verbal consent (this is a double check against the written consent) must be captured. We will be recording the session because we don't want to miss any of your comments. Although one of us may take some notes while we talk, we can't write fast enough to get everything down on paper. As we are recording, please try to speak loudly so that we don't miss your comments.

Themes to be explored

I. Feasibility of implementing the differentiated model of care (motivators and barriers)

Mukora R, *et al. BMJ Open* 2023; 13:e065202. doi: 10.1136/bmjopen-2022-065202

APPENDIX I

STUDY NUMBER: -□□□-□□□□□-□ **AUR2-1-244**

INTERVIEW GUIDE:

DEPARTMENT OF HEALTH KEY STAKEHOLDERS/ FACILITY-BASED STAFF/STUDY STAFF

II. System level challenges of delivering, sustaining and integrating the intervention into the existing TB programme

Time started (HHMM):

Questions

1. **General**

- What is the title of your current position?
- How long have you held this position?
- How are TB services delivered at your level with regard to the intervention? (i.e. District, Provincial, National)
- Please describe your role with the differentiated model of care intervention

2. **Feasibility of implementing the differentiated model of care (motivators and barriers)**

- *Please describe the cadre of staff that were involved with delivering the differentiated model of care?*
- *What are your thoughts on features of the Evrimed device?*
- *Can you describe your experience of delivering the differentiated model of care?*
- *Can you describe the training and resources that staff received prior to or during delivery of the differentiated model of care? What was your opinion of the training and resources received?*
- *Can you describe the benefits of the differentiated model of care and use of the medication device technology?*
- *How often did you follow the requirements for the differentiated model of care?*
- *Please describe the details of what happens on the phone calls and at the home visits.*
- *What tools do you use for the motivational counselling and for adherence counselling?*

Mukora R, *et al. BMJ Open* 2023; 13:e065202. doi: 10.1136/bmjopen-2022-065202

APPENDIX I

STUDY NUMBER: -□□□-□□□□□-□ **AUR2-1-244**

INTERVIEW GUIDE:

DEPARTMENT OF HEALTH KEY STAKEHOLDERS/ FACILITY-BASED STAFF/STUDY STAFF

- *Can you describe the differences between motivational counselling and adherence counselling?*
- *Under what circumstances were not able to follow the requirements for the differentiated model of care?*
- *How can the differentiated model of care be sustained?*
- *Can you describe challenges of the differentiated model of care and use of the medication device technology?*
- *How can TB treatment be improved using this differentiated model of care and the medication device technology?*
- *What was your experience or suggestions regarding interactions with patients? (Giving instructions, or providing motivational counselling)*

3. System level challenges of delivering the intervention

- *How were patients that were part of the differentiated model of care treated differently from those receiving standard of care?*
- *Please elaborate on the positive changes of the differentiated model of care and use of the medication device technology. How do you think these positive changes could be sustained?*
- *Please elaborate on the negative changes of the differentiated model of care and use of the medication device technology. How do you think the negative changes could be addressed?*
- *Can you describe to us what systems are in place that could monitor the differentiated model of care and use of the medication device technology?*
- *Were there any activities that caused or contributed to patients experiencing stigma at the facility? Please elaborate*
- *What types of resources will facilities need to ensure sustainability of the differentiated model of care?*
- *Please describe to us what system level structures need to be improved in order to integrate the differentiated model of care and medication device technology into the existing TB programme system.*

4. Factors that influence the sustainability of the intervention

- *Please describe to us the expectations that you may have had about the differentiated model of care and use of the medication device technology?*

Mukora R, *et al. BMJ Open* 2023; 13:e065202. doi: 10.1136/bmjopen-2022-065202

APPENDIX I

STUDY NUMBER: -□□□-□□□□□-□ **AUR2-1-244**

INTERVIEW GUIDE:

DEPARTMENT OF HEALTH KEY STAKEHOLDERS/ FACILITY-BASED STAFF/STUDY STAFF

- *Please describe your positive and negative experience of delivering or implementing the differentiated model of care.*
- *How different is the current intervention model compared to your initial expectations and how do you think this model could be more effective?*
- *For scale-up and sustainability who do you think should set up the Evrimed boxes for patients? What would be the advantages and disadvantages of this cadre of staff setting up the Evrimed boxes as part of their daily responsibilities.*
- *Can you describe any gaps which exist in the way the intervention is being delivered currently?*

Any other comments

Are there any final thoughts you have about the differentiated model of care and use of the medication device technology?

End of session

Now we have come to the end of our discussion. Thank you for your participation. If you have any questions about your study participation, please contact us. Thank you.

Time ended (HHMM):

Mukora R, *et al. BMJ Open* 2023; 13:e065202. doi: 10.1136/bmjopen-2022-065202
