# Supplementary material for: Qualitative study exploring the feasibility of using medication monitors and a differentiated care approach to support adherence among people receiving TB treatment in South Africa
Source: BMJ Open. 2023 Mar 3;13(3):e065202. doi: 10.1136/bmjopen-2022-065202 (PMC9990642; doi:10.1136/bmjopen-2022-065202)
Supplement: Author's manuscript [file bmjopen-2022-065202.draft_revisions.pdf]

BMJ Open is committed to open peer review. As part of this commitment we make the peer review history of every article we publish publicly available.

When an article is published we post the peer reviewers' comments and the authors' responses online. We also post the versions of the paper that were used during peer review. These are the versions that the peer review comments apply to.

The versions of the paper that follow are the versions that were submitted during the peer review process. They are not the versions of record or the final published versions. They should not be cited or distributed as the published version of this manuscript.

BMJ Open is an open access journal and the full, final, typeset and author-corrected version of record of the manuscript is available on our site with no access controls, subscription charges or pay-per-view fees (http://bmjopen.bmj.com).

If you have any questions on BMJ Open's open peer review process please email info.bmjopen@bmj.com

BMJ Open

"It's more than them just using the box" The feasibility of using medication monitors and a differentiated care approach to improve TB treatment adherence in South Africa

Journal:	*BMJ Open*
Manuscript ID	bmjopen-2022-065202
Article Type:	Original research
Date Submitted by the Author:	30-May-2022

Complete List of Authors:	Mukora, Rachel; The Aurum Institute<br>Maraba, Noriah; The Aurum Institute<br>Orrell, Catherine; University of Cape Town Department of Medicine, Institute of Infectious Disease and Molecular Medicine; Desmond Tutu HIV Foundation<br>Jennings , Lauren; Desmond Tutu HIV Foundation<br>Naidoo, P.; University of Stellenbosch<br>Mbatha, M.; Interactive Research and Development<br>Velen, Kavindhran ; The Aurum Institute<br>Fielding, Katherine; London School of Hygiene & Tropical Medicine, TB Centre; University of the Witwatersrand School of Public Health<br>Charalambous, Salome; The Aurum Institute; University of the Witwatersrand School of Public Health<br>Chetty-Makkan, Candice; Health Economics and Epidemiology Research Office

Keywords:	Public health < INFECTIOUS DISEASES, Tuberculosis < INFECTIOUS DISEASES, Health policy < HEALTH SERVICES ADMINISTRATION & MANAGEMENT, Rationing < HEALTH SERVICES ADMINISTRATION & MANAGEMENT, Organisation of health services < HEALTH SERVICES ADMINISTRATION & MANAGEMENT

SCHOLARONE™
Manuscripts

I, the Submitting Author has the right to grant and does grant on behalf of all authors of the Work (as defined in the below author licence), an exclusive licence and/or a non-exclusive licence for contributions from authors who are: i) UK Crown employees; ii) where BMJ has agreed a CC-BY licence shall apply, and/or iii) in accordance with the terms applicable for US Federal Government officers or employees acting as part of their official duties; on a worldwide, perpetual, irrevocable, royalty-free basis to BMJ Publishing Group Ltd ("BMJ") its licensees and where the relevant Journal is co-owned by BMJ to the co-owners of the Journal, to publish the Work in this journal and any other BMJ products and to exploit all rights, as set out in our licence.

The Submitting Author accepts and understands that any supply made under these terms is made by BMJ to the Submitting Author unless you are acting as an employee on behalf of your employer or a postgraduate student of an affiliated institution which is paying any applicable article publishing charge ("APC") for Open Access articles. Where the Submitting Author wishes to make the Work available on an Open Access basis (and intends to pay the relevant APC), the terms of reuse of such Open Access shall be governed by a Creative Commons licence – details of these licences and which Creative Commons licence will apply to this Work are set out in our licence referred to above.

Other than as permitted in any relevant BMJ Author's Self Archiving Policies, I confirm this Work has not been accepted for publication elsewhere, is not being considered for publication elsewhere and does not duplicate material already published. I confirm all authors consent to publication of this Work and authorise the granting of this licence.

For peer review only

Title: ***"It's more than them just using the box"*** The feasibility of using medication monitors and a differentiated care approach to improve TB treatment adherence in South Africa

Authors: Rachel Mukora[1,8§], Noriah Maraba[1], Catherine Orrell[2,3], Lauren Jennings[3], Pren Naidoo[4], M.Thulani Mbatha[5], Kavindhran Velen[1], Katherine L.Fielding[6,8], Salome Charalambous[1,8], Candice M Chetty-Makkan[7]

Affiliations

1. The Aurum Institute, Aurum House, Parktown, Johannesburg, South Africa
2. University of Cape Town, Department of Medicine, and Institute of Infectious Disease and Molecular Medicine, Cape Town, South Africa
3. Desmond Tutu Health Foundation, Cape Town, South Africa
4. Stellenbosch University, Stellenbosch, South Africa
5. Interactive Research and Development, Durban, South Africa
6. London School of Hygiene and & Tropical Medicine, United Kingdom
7. Health Economics and Epidemiology Research Office, Faculty of Health Sciences, University of the Witwatersrand, Johannesburg, South Africa
8. University of Witwatersrand, School of Public Health, Johannesburg, South Africa

§Corresponding author

Name: Rachel Mukora

Address: The Aurum Institute, Aurum House, The Ridge, 29 Queens Road, Parktown, Johannesburg, 2193, South Africa

Email: rmukora@auruminstitute.org

Keywords

Digital Adherence Technology, Differentiated Care Approach, treatment adherence support, Tuberculosis, Feasibility

Word count abstract: 299

Word count manuscript: 4816

For peer review only - http://bmjopen.bmj.com/site/about/guidelines.xhtml

ABSTRACT

Objectives

The TB MATE study evaluated whether a differentiated care approach (DCA) based on tablet-taking data from Wisepill evriMED digital adherence technology could improve tuberculosis treatment adherence. The DCA entailed a stepwise increase in adherence support starting from short message service (SMS) to phone calls, followed by home visits and motivational counselling. We explored feasibility of this approach with providers in implementing clinics.

Design

Between June 2020 to February 2021, In-depth interviews were conducted in the provider's preferred language, audio recorded, transcribed verbatim and translated. The interview guide included three categories: feasibility, system-level challenges and sustainability of the intervention. We assessed saturation and used thematic analysis.

Setting

Primary healthcare clinics in three provinces of South Africa.

Participants

We conducted 25 interviews with 18 staff and seven stakeholders.

Results

Three major themes emerged: Firstly, providers were supportive of the intervention being integrated into the TB programme and were eager to be trained on the device as it helped to monitor treatment adherence. Secondly, there were challenges in the adoption system such as shortage of human resources which could serve as a barrier to information provision once the intervention is scaled-up. Heath care workers reported that some patients received incorrect SMS's due to delays in the system that contributed to distrust. Thirdly, DCA was considered as a key aspect of the intervention by some staff and stakeholders since it allowed for support based on individual needs.

For peer review only - http://bmjopen.bmj.com/site/about/guidelines.xhtml

Conclusions

It was feasible to monitor tuberculosis treatment adherence using the evriMED device and DCA. To ensure successful scale-up of the adherence support system, emphasis will need to be placed on ensuring that the device and the network operate optimally and continued support on adhering to treatment which will enable people with TB to take ownership of their treatment journey and help overcome TB related stigma.

Strengths and limitations of this study

- We conducted the study in three provinces with different health service characteristics, TB epidemiology and population characteristics which allowed us to understand feasibility of implementation from a broader perspective.
- Another strength was that the study was done in a routine setting with limited resources and without the use of incentives to providers to increase uptake.
- One limitation is that some of the interviews were conducted by a member of the study management team, although this was limited to senior stakeholders who were unlikely to feel reluctant to freely express their views and to withhold some of their opinions.

Key messages

What is already known on this topic

Both globally and in South Africa, there is limited information on the value of Digital Adherence Technologies (DATs) in TB treatment.

What this study adds

Knowledge on the feasibility of using DAT to monitor TB treatment adherence while using a differentiated care approach (DCA) as adherence support for people with TB (PWTB) in South Africa.

How this study might affect research, practice or policy

For peer review only

For peer review only - http://bmjopen.bmj.com/site/about/guidelines.xhtml

This research shows that technology on its own will not solve treatment adherence issues especially those related to stigma and lack of support. Sound communication between PWTB and providers serves as a key tool to improving treatment outcomes.

INTRODUCTION

Globally the treatment success rate for new and relapse drug-sensitive TB (DS-TB), is 86% while in South Africa (SA) this is much lower at 79%, 78% for those who are HIV positive with TB and 67% for those previously treated with TB(1). Treatment failure is often a result of non-adherence to treatment, loss to follow-up or unevaluated outcomes(2). Typical reasons that people with TB (PWTB) might not adhere to treatment include false perceptions of being cured once they feel better; stigma; forgetfulness; lack of social network support; and poor user experience of accessing care at clinics(3). Traditional methods such as Directly Observed Treatment Short course (DOTs), pill counts and self-report have limitations and have not been shown to improve treatment adherence in PWTB(4),(5). Digital adherence technology (DAT) including medication monitors may overcome challenges to monitoring tuberculosis treatment adherence through remotely documenting dosing patterns of PWTB. DATs have been recommended by the World Health Organization (WHO) based on evidence from a study in China where drug-sensitive TB patients received an electronic medication monitor to support self-administered treatment for TB(6).

Despite this recommendation, both globally and in South Africa, there is limited information on the value of DATs in TB treatment. A study done in KwaZulu-Natal SA amongst drug resistant TB-HIV co-infected inpatients using an older version of the Wisepill device, the RT2000 3G, showed that feasibility challenges for digital pillboxes may include battery failure, device malfunction and problems related to cellular networks(7). Previous studies have found that other DAT such as SMS reminders for TB treatment were insufficient to improve treatment adherence alone. Barriers included frequent changing of phone numbers

For peer review only - http://bmjopen.bmj.com/site/about/guidelines.xhtml

and uncertainty whether patients were taking their tablets immediately after they received the SMS or not(8). Another study done in Uganda for HIV treatment showed that challenges for cellphone-based strategies included: use of shared cell phones, technical failures preventing receipt of SMS texts, electricity outages and changing phone numbers(9, 10).

Most DATs have focused on monitoring adherence and few studies have used DATs to differentiate between those patients who are struggling and need individual support, and those who are doing well. Given this gap in knowledge, we evaluated the feasibility of using the Wisepill evriMED DAT to monitor TB treatment adherence while using a differentiated care approach (DCA) with a stepwise increase in adherence support for PWTB in South Africa.

METHODS

Study design and study setting

This qualitative study was embedded within a cluster-randomized trial (CRT) that took place in six clinics in each of three provinces of South Africa: Gauteng (Ekurhuleni district); Western Cape (Klipfontein and Mitchell's Plain districts); and Kwa-Zulu Natal (eThekwini district), the details of which have been published elsewhere(11). In the intervention arm of the CRT, PWTB received medication monitors with reminders triggering differentiated care approach (DCA) in response to adherence data uploads, carried out from a central database(11). The DCA was implemented in a progressive manner depending on the number of doses a participant missed(11). If one dose was missed, then a short message service (SMS) reminder was sent to the participant(11). If a second or third dose was missed, then study staff would make a telephone call to the participant and once the fourth dose was missed then a home visit was conducted during which motivational counselling took place(11). We describe the *feasibility* of implementing Wisepill evriMED device and differentiated care from a stakeholder perspective.

For peer review only - http://bmjopen.bmj.com/site/about/guidelines.xhtml

Site selection

There were a total of nine intervention clinics, three in each province. Clinics were selected based location, HIV prevalence and numbers of patients starting TB treatment per month(11).

Study population

The study population consisted of seven purposively selected stakeholders from the Department of Health (DOH): one national, one provincial and five district-level representatives, who worked closely with the facilities using the electronic device (Wisepill evriMED DAT). From each intervention facility, we interviewed one facility staff (government employee) and one study staff member. All study staff worked on the project for at least three months. Facility staff-initiated patients on TB treatment and monitored patients for their scheduled monthly follow-up visits while study staff offered PWTB the device, followed-up on those who had missed doses and provided motivational and adherence counselling.

Patient and public involvement

Patients or the public were not involved in the design, or conduct, or reporting, or dissemination plans of our research.

Conceptual framework and themes explored

Using the feasibility framework suggested by Bowen et al(12), we developed an in depth interview guide that covered the following topics; (i) the relative ease of implementation and operation of the technology within existing health systems, technology infrastructure and supply chain; (ii) system level challenges of delivering, sustaining and integrating the intervention into the existing TB programme. We used the framework on the ‘Integration and sustainability of interventions into health systems’(13) to organize the data.

For peer review only - http://bmjopen.bmj.com/site/about/guidelines.xhtml

Data collection

We conducted a total of 25 in-depth interviews. The sample was representative of providers involved in the intervention while also considering saturation where themes usually start to converge after 15 interviews. The IDI guide was piloted with one stakeholder, two facility and one study staff between January to February 2020. The probing questions were adapted after the pilot. These interviews were conducted face-to-face, and the data was included in the analysis. Data collection continued between June 2020 to January 2021 with each interview lasting between 45 – 90 minutes. The interviews were all conducted in the providers preferred language by a female PhD student (RM) and a female study coordinator who was a PhD student with qualitative experience (VM). Both researchers established a prior relationship with the providers enabling them to understand the reasons for the study. The interviews were digitally recorded with the providers consent and transcribed verbatim by trained research assistants (Masters students). The transcripts were not returned to the providers for comment or correction. Due to the impact of COVID-19 and the national lockdown that took place, these interviews were conducted virtually over Microsoft Teams. No one else was present in the interviews besides the providers and the researchers and field notes were made during the interviews. Saturation was assessed during data collection through asking the same question in different ways and reviewing a sample of the recordings until no new information was obtainable. No repeat interviews were carried out.

Data analysis

Thematic analysis was used with deductive and inductive approaches(14, 15). At least 10% of the transcripts were coded inductively by two independent researchers so as to reduce bias and improve reliability(14). A codebook of emerging themes was developed, guided by the framework on the 'Integration and sustainability of interventions into health systems' (13),with discrepancies being resolved through discussion. Where there was no inter-rater agreement, the theme was dropped(14). The final codebook was used to code the remaining transcripts and any new codes that emerged were also included in this codebook. MAXQDA

For peer review only

qualitative software was used for the coding process. We highlight the major themes and soft themes that emerged and use supportive direct quotations from providers. Providers did not provide feedback on the findings.

RESULTS

We conducted 25 interviews in total with 18 staff (9 facility staff and 9 study staff) and 7 stakeholders (1 national,1 provincial and 5 at district-level) across 3 provinces. Majority of the providers (23 out of 25) were female. None of the approached providers declined to participate. Major themes included (1) Providers were supportive of the intervention, (2) Intervention challenges within the adoption system and broad context, and (3) Ensuring intervention sustainability through constant training of staff and education of PWTB

Main Theme 1: Providers were supportive of the intervention

There was buy-in from staff who felt involved and were supportive of the intervention. Stakeholders were also very supportive of the intervention despite their limited involvement.

> *"… to me it was a very good idea and it was working very well … it improved our cure rate because we had a lot of patients who were defaulting and who and to be referred to the hospital because they developed multi, MDR. So since we had …this intervention, at least we had less patients who developed multi-drug resistance."*
>
> *- Female, facility staff*

> *"…I think…it's magnificent. I think it's… a tool box, it's really a way for us to see what's really happening at the point of … TB treatment, where the patient take… the medication when they open the box. So I think it's really innovation that can be used. ... I think it also is a reminder for the patient. You know? That … "I need to take my medication". And it's a way to …to ensure the quality of the programme, that patients*

For peer review only - http://bmjopen.bmj.com/site/about/guidelines.xhtml

are adhering to the ... medication…I think it is a critical intervention." – Female, DOH stakeholder (District level)

Both facility and study staff found the device easy to learn and facility staff who had received a briefing on the study were eager to receive the complete training to support the study staff.

"Because sometimes she (referring to study staff) [might not be here and it happens that she's not here, either she is sick or she's gone on holiday for December. She says: "…we can show you and then you do this things when we are not here". I said: "… it will be easy… As long as you show us, we can do that device".

- Female, facility staff

The staff described the activities of the intervention such as issuing devices, phone calls and making home visits as being well integrated into the TB programme. Providers had strong support for the device for two main reasons that we classified as sub-themes in our analysis:

Sub-theme 1: Device as a useful reminder and early notification tool

Most providers found the alarm that was fitted on the device to be a useful reminder to patients to take their treatment on time and to attend their clinic visits. The device also alerted providers to PWTB who were not using the device and not taking treatment.

"I feel like it assists with the real time monitoring because now if you check on the system, you would be able to check instead of waiting like someone waiting for their appointment that is months after, you can actually check now on the system that this person there's no activity, let me call and you would actually find that the person has died or maybe the person has- is in hospital is admitted.."- Female, study staff

For peer review only - http://bmjopen.bmj.com/site/about/guidelines.xhtml

"So, it will help us because …sometimes we notice the other patient…didn't take their medication and start to recall all of them and then the other just default … With the box, someone is getting … a notification that this person is not taking her treatment..." – Female, facility staff

However, some staff distrusted that opening of the device meant that PWTB took their pills.

"…some patients do that when they open the pill box and … show an adherence of 100%, but still they… have not been taking their medications. So we would try to motivate them, counsel them, explain the disadvantages of not of them not taking the medication." – Female, study staff

"I think because you know patients always find a way to find loopholes within the health system, so at some point they will understand that you're recording the opening and the closing of the box not necessarily them actively taking the medications. So they can easily open and close the box without really taking the medication." – Female, DOH stakeholder (District level)

Sub-theme 2: Differentiated Care Approach allows for patient-centred care

Staff mentioned that they found DCA to be a positive and unique aspect of our intervention.

"So, you are no longer administering intervention for one. You sort of trying to be specific …to a person and offer them care in their in their specific sort of situations. So, I think that is a positive thing because you don't assume everyone is the same. You don't assume everyone's situation is the same. You understand that you are dealing with … individuals. That's … what I think is positive about this differentiated model of care." – Male, study staff

For peer review only - http://bmjopen.bmj.com/site/about/guidelines.xhtml

"… yah, I think I think the differentiated model of care … is a very positive thing, that it works to an extent because it's not a blanket approach. You don't, you don't think of every patient as the same…you sort of attend to each person in their context and … try to understand what they are going through." – Male, study staff

The staff also felt that the counselling they provided as part of the DCA helped them to understand their patients' reasons for not taking their treatment thus allowing them to manage them better as they supported them on their treatment journey.

"This whole approach I feel like it's a great initiative because it assists in managing the patient - like fully. Like if I can say wholly, not just managing the patient, taking the medication. You could also like… with the counselling you actually find that the problem is that the participant is facing beyond treatment intake."- Female, study staff

Phone calls to PWTB allowed for clarification on any issues that may not have been clear to the patient while at the clinic.

"…he didn't understand but when I called him and I was speaking to him over the phone to understand how they're taking the treatment, I find that … yeah there was mistake … one of the best things about this intervention because some of them are able to even call me up, I don't always now have to call patients."

– Male, study staff

Home visits allowed the staff to have better interaction with their patients and to build trust with them making them feel like someone cares about them.

For peer review only - http://bmjopen.bmj.com/site/about/guidelines.xhtml

"It's not bad. It's nice to do home visits. It's whereby … you contact with the patients, know each other … where the patient has got the problem, it's with the … problem with the box or she wanted to ask me something that she forget to ask at the clinic." – Female, study staff

"… I think the participant at least get that thing when you do home visits, or you phone them when they are not taking the treatment they feel as if they are cared of, someone is care is cared for me when I am not taking the treatment because I would get a call, or I will get a home visit''- Female, study staff

Some providers described DCA as more of a journey of supporting the patients and helping build trust between the staff and the PWTB.

"… that is really good part of the intervention. I think it … continue to build that trust…And that support for the patients … so I- I think that was impressive." - Female, DOH stakeholder (District level)

"I thought it was only about the box… it's more than them just using the box. It's their treatment journey and being supported to adhere to the treatment and get healed from the TB." – Female, study staff

Major Theme 2: Intervention challenges within the adoption system and broad context

Some staff felt that the trust they built with the PWTB could be threatened by human resource shortages and a dedicated cadre was needed to ensure successful implementation.

"However, when we find at once the research study stop and we over onto implementation, … the constraints is always HR resources and at the operational level, if that initial education isn't framed correctly. There could be a

For peer review only - http://bmjopen.bmj.com/site/about/guidelines.xhtml

misunderstanding about the purpose of the box." - Female, DOH stakeholder (District level)

Challenges that were encountered with the device or the network resulted in delays in the system updating that the box was opened which staff felt created trust issues between them and the participant.

"Now it's creating a trust issue between you and the patient, because you phoning regarding treatment that was not taken because … Wisepill says treatment was not taken, while the patient on the other side did take the medication." - Female, study staff

Heath care workers reported that some patients received incorrect SMS's due to delays in the system.

'' Especially when it has been a weekend and then they will not … send out the data of… the weekend like … if there were any missed or any … intakes. Sometimes they will show that they missed the weekend and they did not take the medication, but in three days down the line… the correct information will show. That's where we will see that there were no missed doses.''- Female, study staff

Staff also felt that that the device would not work for some group of patients such as those who abuse substances.

"The patient that are on substance abuse, yoh! You know, when they come here, they are ok. At a later stage, once they've started treatment, you realise that this patient is using substances…With those patients, they the device- ya. It's not good. Won't- won't work. Won't- won't help." – Female, facility staff

For peer review only - http://bmjopen.bmj.com/site/about/guidelines.xhtml

"He took the evriMED box device and the TB meds. That was the last time we saw him on the clinic. He run away from home apparently. He was on drugs. But then…he is back. He's here in the TB room. He's got TB again. So I was asking him the other day: "are you still interested in joining this study?". And he was like: "you know what I did with the first box? I sold it". - Female, study staff

Sub-theme 3: Provider perceptions of stigma related to use of the evriMED device and DCA

Staff perceived that some of the features of the box such as the alarm and the size of the box may have been a concern to some PWTB in trying to conceal their TB status so they would opt to leave their devices behind when travelling on holiday or going to work for fear of disclosure of their TB status.

"The thing that is common, especially in December … an example is person supposed to take it at 8, they take it at 8 but they not taking it at 8 from the box... I think they went for holidays … This person is taking treatment and the putting it… in a purse. Whereby you'll take in the morning without this alarm ringing.''- Male, study staff

"So some will come to us and say: "what if the pill box doesn't make so much noise? It will be easier for me to carry around- or if it was a bit … smaller, then it will be easier for me to carry it. But now it's big, I don't want … people seeing me like with this pill box in the taxi or at work."- Female, study staff

"I haven't disclosed to my partner that I'm on TB medication and imagine if I had to carry this box and the box would be ringing in the morning and it would cause unnecessary fights." - Female, study staff.

For peer review only - http://bmjopen.bmj.com/site/about/guidelines.xhtml

Providers perceived that stigma was more of an issue outside the PWTB household as often they would not disclose their TB status to members outside their homes.

> *"So, I noticed that it's comfortable for them to use this box when they are at home with the people that actually know what is going on with them but when they are going to other people that are not aware that they are sick or anything, it seems as if they don't want to carry the boxes." - Female, study staff.*

Staff perceived that stigma related to DCA existed in the community and some of them witnessed the PWTB fear of being stigmatized when conducting home visits. PWTB would often ask staff not to wear their uniforms when they visit their homes.

> *"Uhm they hate it when we wear our TB MATE t-shirts, because they are saying: "due to stigma". When we go visit their homes, we must not wear something that will be written 'TB' or 'HIV'. So we wear our normal clothes..."- Female, study staff.*

> *"Then I took off my jacket, and then the participant saw that TB MATE on my t-shirt. And then the patient told me that I must wear my jacket because what if someone walks in and sees the TB MATE on my t-shirt. So I wasn't aware that the participant didn't disclose... She told me that she's got fear that- because she will be judged because she was drinking alcohol a lot. But now she's got TB. They will say she must go and stay in the back room."- Female, study staff*

Staff perceived that some stigma could have been internalised and not actually experienced by PWTB.

For peer review only - http://bmjopen.bmj.com/site/about/guidelines.xhtml

"It's their thoughts because the person would feel like it did not want to be seen carrying the box. Because people will think- so he sees all their thoughts. It's not something that really happened."- Female, facility staff

"But in the clinic, there wasn't really anything that contributed to the stigma but maybe with now giving the pill box… some of the patients would say that maybe it's gonna show. Maybe with their ARV's they could take it in private. Now we have them having this pill box that is gonna ring even, that is gonna beep and remind them to take treatment..'' – Female, study staff

Major Theme 3: Ensuring intervention sustainability through constant training of staff and education of PWTB

Education on the intervention emerged as one of the major themes that stakeholders and staff placed emphasis on as a means of managing their patients in a holistic way leading to successful treatment outcomes. Education and training should emphasize the importance of adhering to treatment and should be strengthened at various levels – with the PWTB themselves, those who would be involved in implementation such as community health workers (CHWs) and also to the community at large.

''My experience was that as the community we need to need to educate the community the importance of complying to medication…we need to educate and educate and educate.''- Female, facility staff

"But I definitely think it's… strengthening that interface between the patient and the staff... it's an important interface. And- and of course that too- the 'how' of- of the contact, if it is about 'why you didn't- why you aren't taking your treatment' rather than saying 'how are you doing'- you know- 'are you ok', uhm 'how can I help you'. When we start off with those different conversations, it can lead to different outcomes."

For peer review only - http://bmjopen.bmj.com/site/about/guidelines.xhtml

– Female, DOH stakeholder (District level)

The staff felt that constant education and reinforcement would ultimately lead to behaviour change and treatment adherence.

"I think the intervention even though we are the one offering the intervention but we sort of gave patients the power to understand the… reasons why they are taking treatment, why is it important for them to…adhere to the treatment…we were actually able to make patients to take their treatment journey into their own hands … it's almost like we were empowering patients…"- Male, study staff

DISCUSSION

Using the Wisepill evriMED device to support adherence to TB treatment appears to be a feasible option in South Africa. Providers were supportive of the evriMED device and the differentiated care approach, as they felt it had a positive impact on the TB patient's programme. Stakeholders were supportive despite their involvement being limited to approval of the study and providing oversight of the TB programme.

This study has shown that DATs is an innovative approach that might improve the management of PWTB by allowing individual differentiated support. Early notification of missed doses from the device allowed staff to intervene through SMS messaging and phone calls, thus reducing the need for a home visit. This freed up time for staff to focus on other duties. These findings are similar to other studies where differentiated care models used in HIV treatment delivery have addressed challenges such as overcrowded facilities, overburdened staff and long waiting times in countries like Uganda, Swaziland, Mozambique and South Africa(6, 16-18).

For peer review only

Staff noted that the differentiated care allowed a journey of educating and supporting PWTB. Through adherence and motivational counselling, staff empowered patients to understand the importance of adhering to treatment and to empower them to take their treatment journey into their own hands. For scale-up, the real time monitoring feature of the device is important so that the correct individuals are promptly identified for further support before they become lost to follow-up. This also allows for the efficient utilisation of resources to those in most need, as seen in various studies(4, 19, 20). More attention should also be given to relationship training as the sustainability of the intervention depends on a sound provider-patient relationship. Correct framing of the intervention as a support tool would also help ensure that the intervention is well perceived once scaled-up. Training needs to be constant and not once-off to reinforce their understanding of the intervention.

Some challenges with the technology were cited such as alarm malfunction, incorrectly sent SMS's and short battery lifespan. These challenges created distrust from PWTB, and the device would therefore need to be of the highest quality to ensure sustainability of the intervention. A systematic review of DAT for the management of tuberculosis therapy found similar feasibility challenges remain in low- and middle-income settings(9).Once the intervention is scaled up, the shortage of human resources (HR) could serve as a barrier to ensuring that providers receive the correct information. For successful scale-up, some staff recommended that a dedicated cadre should be in place to follow-up PWTB to ensure that they understand the importance of taking treatment. However, since the support system had reduced the need for home visits and follow up calls, the current TB cadres, clerical staff and community-based teams were also seen as being sufficient. Despite any challenges experienced within the adoption system and broad context, the staff were all very supportive of the intervention and even recommended that it should be used for all TB and chronic patients as well as in other facilities.

For peer review only - http://bmjopen.bmj.com/site/about/guidelines.xhtml

Fear of stigma and the disclosure of one’s TB status has the potential to act as a barrier to scale-up and sustainability of the intervention, if not well addressed. Stigma and fear of disclosure resulting in lower acceptance of DAT technologies amongst multi-drug resistant TB patients have also been reported in India(21), with improvements recommended on the design. Disease-related stigma may be more difficult to address, and screening should be used to identify upfront those patients whom stigma and fear of disclosure may limit the use of the MERM(21).

A limitation of the study was that interviews had to be conducted virtually on Microsoft teams, due to the Covid-19 pandemic, and to allow for effective social distancing for both the researchers and the study providers. This new mode of conducting interviews had setbacks such as network and connectivity challenges which interrupted the flow of some of the interviews. The interviewers mitigated this challenge through re-iterating what the participant had said to ensure that the correct message had been captured. Through the consenting process, we were able to ensure confidentiality and that the participant was relaxed. A second limitation is that some of the interviews were conducted by a member of the study management team, although this was limited to senior stakeholders who were unlikely to feel reluctant to freely express their views and to withhold some of their opinions. We conducted the study in three provinces with different health service characteristics, TB epidemiology and population characteristics which allowed us to understand feasibility of implementation from a broader perspective. Another strength was that the study was done in a routine setting with limited resources and without the use of incentives to providers to increase uptake.

CONCLUSION

Digital adherence technology (DAT) such as medication monitors have a huge potential to improve tuberculosis treatment adherence. However, to ensure successful scale-up and

For peer review only - http://bmjopen.bmj.com/site/about/guidelines.xhtml

sustainability of the intervention, the differentiated care approach should be used as a platform to constantly educate PWTB and the community at large on TB and the importance of adhering to treatment since technology on its own will not solve treatment adherence issues especially those related to stigma and lack of support. Sound communication between PWTB and providers serves as a key tool to improving treatment outcomes thus more attention should be given to relationship training to ensure that the provider-patient relationships provide the necessary support to those who need it most.

ABBREVIATIONS

COVID	Corona Virus Disease
DCA	Differentiated Care Approach
DOH	Department of Health
DS-TB	Drug-sensitive TB
DATs	Digital Adherence Technologies
DOTs	Directly Observed Treatment Short Course
ICFs	Informed Consent Forms
IDI	In-depth Interview
MDR-TB	Multidrug-resistant TB
PWTB	People With TB
SA	South Africa
SMS	Short Message Service
TB	Tuberculosis
TB MATE	TB Monitoring Adherence to Treatment Endpoints

ACKNOWLEDGEMENTS

We would like to thank the following: Ekurhuleni, City of Cape Town, eThekwini districts and the Ekurhuleni Health District Research Committee (EHDRC) for allowing us to conduct the study in their districts. The study coordinators from Gauteng, Western Cape and KwaZulu-Natal in South Africa for their assistance with data collection namely Israel Rabothata, Vuyelwa Mehlomakulu (VM), Vumile Gumede and Bongani Zondi and their field-based teams of research assistants and interns; all the transcribers who dedicated time to capture the conversations verbatim; all the providers for taking part in this study

CONTRIBUTORSHIP STATEMENT

RM, NM, LJ, MTM, KLF, SC and CMCM conceptualized the study. RM and CMCM analysed the data. RM, NM, CO, SC and CMCM interpreted the data. RM wrote the original draft and revised subsequent versions of the manuscript. SC and CMCM provided guidance on earlier versions of the manuscript. NM, CO, LJ, PN, MTM, KV, KLF, SC and CMCM reviewed and

For peer review only

For peer review only - http://bmjopen.bmj.com/site/about/guidelines.xhtml

544 edited previous versions. RM, SC and CMCM are responsible for the overall content of the
545 manuscript. The authors read and approved the final manuscript.

546 COMPETING INTERESTS

547 The authors declare that they have no competing interests.

548 FUNDING STATEMENT

549 The study is funded by (1) Bill & Melinda Gates Foundation (OPP1205388), (2) TB REACH
550 Wave 6 project of Stop TB Partnership (STBP/TBREACH/GSA/W6-34), and (3) South
551 African Medical Research Council through the Strategic Health Innovation Partnerships.
552 Funders did not play a part in the design of the study or the decision to submit the
553 manuscript for publication.

554 DATA SHARING STATEMENT

555 No additional data available.

556 ETHICS APPROVAL AND PARTICIPANT CONSENT

557 The parent study received ethics approval from the three district and provincial ethics
558 committees where the trial sites were located. This qualitative study was approved by the
559 Human Research Ethics Committee (HREC) of the University of Witwatersrand (Ref 180705)
560 and the University of Cape Town (Ref 452/2018). We obtained written informed consent for
561 study participation and permission for digital recording from all providers. Providers were not
562 reimbursed.

563 REFERENCES

564 1. WHO. Global Tuberculosis Report 2021. Geneva: World Health Organization 2021 [Available
565 from:
566 https://worldhealthorg.shinyapps.io/tb_profiles/?_inputs_&entity_type=%22country%22&lan=%22E
567 N%22&iso2=%22ZA%22.
568 2. WHO. Global Tuberculosis Report 2018. Geneva: World Health Organization.
569 3. Cramm JM, Finkenflugel HJ, Moller V, Nieboer AP. TB treatment initiation and adherence in a
570 South African community influenced more by perceptions than by knowledge of tuberculosis. BMC
571 Public Health. 2010;10:72.
572 4. Serapelwane MG, Davhana-Maselesele M, Masilo GM. Experiences of patients having
573 tuberculosis (TB) regarding the use of Directly Observed Treatment Short-Course (DOTS) in the North
574 West Province, South Africa. Curationis. 2016;39(1):e1-e9.
575 5. Knight M, van Zyl RL, Sanne I, Bassett J, van Rie A. Impact of combination antiretroviral
576 therapy initiation on adherence to antituberculosis treatment. South Afr J HIV Med. 2015;16(1):346.
577 6. Liu X, Lewis JJ, Zhang H, Lu W, Zhang S, Zheng G, et al. Effectiveness of Electronic Reminders
578 to Improve Medication Adherence in Tuberculosis Patients: A Cluster-Randomised Trial. PLoS Med.
579 2015;12(9):e1001876.
580 7. Bionghi N, Daftary A, Maharaj B, Msibi Z, Amico KR, Friedland G, et al. Pilot evaluation of a
581 second-generation electronic pill box for adherence to Bedaquiline and antiretroviral therapy in

For peer review only - http://bmjopen.bmj.com/site/about/guidelines.xhtml

drug-resistant TB/HIV co-infected patients in KwaZulu-Natal, South Africa. BMC Infect Dis. 2018;18(1):171.

8. Hüsler J. Evaluation of the On Cue Compliance Service Pilot : Testing the use of SMS reminders in the treatment of Tuberculosis in Cape Town, South Africa. 2005.

9. Subbaraman R, de Mondesert L, Musiimenta A, Pai M, Mayer KH, Thomas BE, et al. Digital adherence technologies for the management of tuberculosis therapy: mapping the landscape and research priorities. BMJ Glob Health. 2018;3(5):e001018.

10. Hermans SM, Elbireer S, Tibakabikoba H, Hoefman BJ, Manabe YC. Text messaging to decrease tuberculosis treatment attrition in TB-HIV coinfection in Uganda. Patient Prefer Adherence. 2017;11:1479-87.

11. Maraba N, Orrell C, Chetty-Makkan CM, Velen K, Mukora R, Page-Shipp L, et al. Evaluation of adherence monitoring system using evriMED with a differentiated response compared to standard of care among drug-sensitive TB patients in three provinces in South Africa: a protocol for a cluster randomised control trial. Trials. 2021;22(1):389.

12. Bowen DJ, Kreuter M, Spring B, Cofta-Woerpel L, Linnan L, Weiner D, et al. How we design feasibility studies. Am J Prev Med. 2009;36(5):452-7.

13. Atun R, de Jongh T, Secci F, Ohiri K, Adeyi O. Integration of targeted health interventions into health systems: a conceptual framework for analysis. Health Policy Plan. 2010;25(2):104-11.

14. Pope C, Ziebland S, Mays N. Qualitative research in health care. Analysing qualitative data. BMJ. 2000;320(7227):114-6.

15. Charmaz K. Constructing Grounded Theory: A practical guide through qualitative analysis Nurse Res. 2006;13(4):84.

16. Wilkinson L, Harley B, Sharp J, Solomon S, Jacobs S, Cragg C, et al. Expansion of the Adherence Club model for stable antiretroviral therapy patients in the Cape Metro, South Africa 2011-2015. Trop Med Int Health. 2016;21(6):743-9.

17. Decroo T, Telfer B, Biot M, Maikere J, Dezembro S, Cumba LI, et al. Distribution of antiretroviral treatment through self-forming groups of patients in Tete Province, Mozambique. J Acquir Immune Defic Syndr. 2011;56(2):e39-44.

18. Okoboi S, Ding E, Persuad S, Wangisi J, Birungi J, Shurgold S, et al. Community-based ART distribution system can effectively facilitate long-term program retention and low-rates of death and virologic failure in rural Uganda. AIDS Res Ther. 2015;12:37.

19. Kurz SG, Furin JJ, Bark CM. Drug-Resistant Tuberculosis: Challenges and Progress. Infect Dis Clin North Am. 2016;30(2):509-22.

20. Subbaraman R, Nathavitharana RR, Mayer KH, Satyanarayana S, Chadha VK, Arinaminpathy N, et al. Constructing care cascades for active tuberculosis: A strategy for program monitoring and identifying gaps in quality of care. PLoS Med. 2019;16(2):e1002754.

21. Thomas BE, Kumar JV, Periyasamy M, Khandewale AS, Hephzibah Mercy J, Raj EM, et al. Acceptability of the Medication Event Reminder Monitor for Promoting Adherence to Multidrug-Resistant Tuberculosis Therapy in Two Indian Cities: Qualitative Study of Patients and Health Care Providers. J Med Internet Res. 2021;23(6):e23294.

For peer review only

For peer review only - http://bmjopen.bmj.com/site/about/guidelines.xhtml

BMJ Open

"It's more than them just using the box" A qualitative study exploring the feasibility of using medication monitors and a differentiated care approach to support adherence among people receiving TB treatment in South Africa

Journal:	*BMJ Open*
Manuscript ID	bmjopen-2022-065202.R1
Article Type:	Original research
Date Submitted by the Author:	27-Jan-2023
Complete List of Authors:	Mukora, Rachel; The Aurum Institute<br>Maraba, Noriah; The Aurum Institute<br>Orrell, Catherine; University of Cape Town Department of Medicine, Institute of Infectious Disease and Molecular Medicine; Desmond Tutu HIV Foundation<br>Jennings , Lauren; Desmond Tutu HIV Foundation<br>Naidoo, P.; University of Stellenbosch<br>Mbatha, M.; Interactive Research and Development<br>Velen, Kavindhran ; The Aurum Institute<br>Fielding, Katherine; London School of Hygiene & Tropical Medicine, TB Centre; University of the Witwatersrand School of Public Health<br>Charalambous, Salome; The Aurum Institute; University of the Witwatersrand School of Public Health<br>Chetty-Makkan, Candice; Health Economics and Epidemiology Research Office
**Primary Subject Heading**:	Qualitative research
Secondary Subject Heading:	Public health, Global health
Keywords:	Public health < INFECTIOUS DISEASES, Tuberculosis < INFECTIOUS DISEASES, Health policy < HEALTH SERVICES ADMINISTRATION & MANAGEMENT, Rationing < HEALTH SERVICES ADMINISTRATION & MANAGEMENT, Organisation of health services < HEALTH SERVICES ADMINISTRATION & MANAGEMENT

SCHOLARONE™
Manuscripts

I, the Submitting Author has the right to grant and does grant on behalf of all authors of the Work (as defined in the below author licence), an exclusive licence and/or a non-exclusive licence for contributions from authors who are: i) UK Crown employees; ii) where BMJ has agreed a CC-BY licence shall apply, and/or iii) in accordance with the terms applicable for US Federal Government officers or employees acting as part of their official duties; on a worldwide, perpetual, irrevocable, royalty-free basis to BMJ Publishing Group Ltd ("BMJ") its licensees and where the relevant Journal is co-owned by BMJ to the co-owners of the Journal, to publish the Work in this journal and any other BMJ products and to exploit all rights, as set out in our licence.

The Submitting Author accepts and understands that any supply made under these terms is made by BMJ to the Submitting Author unless you are acting as an employee on behalf of your employer or a postgraduate student of an affiliated institution which is paying any applicable article publishing charge ("APC") for Open Access articles. Where the Submitting Author wishes to make the Work available on an Open Access basis (and intends to pay the relevant APC), the terms of reuse of such Open Access shall be governed by a Creative Commons licence – details of these licences and which Creative Commons licence will apply to this Work are set out in our licence referred to above.

Other than as permitted in any relevant BMJ Author's Self Archiving Policies, I confirm this Work has not been accepted for publication elsewhere, is not being considered for publication elsewhere and does not duplicate material already published. I confirm all authors consent to publication of this Work and authorise the granting of this licence.

For peer review only

Title: ***"It's more than them just using the box"*** A qualitative study exploring the feasibility of using medication monitors and a differentiated care approach to support adherence among people receiving TB treatment in South Africa

Authors: Rachel Mukora[1,8§], Noriah Maraba[1], Catherine Orrell[2,3], Lauren Jennings[3], Pren Naidoo[4], M.Thulani Mbatha[5], Kavindhran Velen[1], Katherine L.Fielding[6,8], Salome Charalambous[1,8], Candice M Chetty-Makkan[7]

Affiliations

1. The Aurum Institute, Aurum House, Parktown, Johannesburg, South Africa
2. University of Cape Town, Department of Medicine, and Institute of Infectious Disease and Molecular Medicine, Cape Town, South Africa
3. Desmond Tutu Health Foundation, Cape Town, South Africa
4. Stellenbosch University, Stellenbosch, South Africa
5. Interactive Research and Development, Durban, South Africa
6. London School of Hygiene and & Tropical Medicine, United Kingdom
7. Health Economics and Epidemiology Research Office, Faculty of Health Sciences, University of the Witwatersrand, Johannesburg, South Africa
8. University of Witwatersrand, School of Public Health, Johannesburg, South Africa

§Corresponding author

Name: Rachel Mukora

Address: The Aurum Institute, Aurum House, The Ridge, 29 Queens Road, Parktown, Johannesburg, 2193, South Africa

Email: rmukora@auruminstitute.org

Keywords

Digital Adherence Technology, Differentiated Care Approach, treatment adherence support, Tuberculosis, Feasibility

Word count abstract: 299

Word count manuscript: 4816

1

For peer review only

ABSTRACT

Objectives

The TB MATE study evaluated whether a differentiated care approach (DCA) based on tablet-taking data from Wisepill evriMED digital adherence technology could improve tuberculosis treatment adherence. The DCA entailed a stepwise increase in adherence support starting from short message service (SMS) to phone calls, followed by home visits and motivational counselling. We explored feasibility of this approach with providers in implementing clinics.

Design

Between June 2020 to February 2021, In-depth interviews were conducted in the provider's preferred language, audio recorded, transcribed verbatim and translated. The interview guide included three categories: feasibility, system-level challenges and sustainability of the intervention. We assessed saturation and used thematic analysis.

Setting

Primary healthcare clinics in three provinces of South Africa.

Participants

We conducted 25 interviews with 18 staff and seven stakeholders.

Results

Three major themes emerged: Firstly, providers were supportive of the intervention being integrated into the TB programme and were eager to be trained on the device as it helped to monitor treatment adherence. Secondly, there were challenges in the adoption system such as shortage of human resources which could serve as a barrier to information provision once the intervention is scaled-up. Heath care workers reported that some patients received incorrect SMS's due to delays in the system that contributed to distrust. Thirdly, DCA was considered as a key aspect of the intervention by some staff and stakeholders since it allowed for support based on individual needs.

For peer review only - http://bmjopen.bmj.com/site/about/guidelines.xhtml

Conclusions

It was feasible to monitor tuberculosis treatment adherence using the evriMED device and DCA. To ensure successful scale-up of the adherence support system, emphasis will need to be placed on ensuring that the device and the network operate optimally and continued support on adhering to treatment which will enable people with TB to take ownership of their treatment journey and help overcome TB related stigma.

Strengths and limitations of this study

- We conducted the study in three provinces with different health service characteristics, TB epidemiology and population characteristics which allowed us to understand feasibility of implementation from a broader perspective.
- Another strength was that the study was done in a routine setting with limited resources and without the use of incentives to providers to increase uptake.
- One limitation is that some of the interviews were conducted by a member of the study management team, although this was limited to senior stakeholders who were unlikely to feel reluctant to freely express their views and to withhold some of their opinions.

INTRODUCTION

Globally the treatment success rate for new and relapse drug-sensitive TB (DS-TB), is 86% while in South Africa (SA) this is much lower at 79% [1]. Amongst those who are HIV positive with TB, the treatment success rate is 78% and 67% for those previously treated with TB [1]. Treatment failure is often a result of non-adherence to treatment, loss to follow-up or unevaluated outcomes [2]. Typical reasons that people with TB (PWTB) might not adhere to treatment include false perceptions of being cured once they feel better, stigma, forgetfulness, lack of social network support, and poor user experience of accessing care at clinics [3]. Traditional methods such as Directly Observed Treatment Short course (DOTs), pill counts and self-report have limitations and have not been shown to improve treatment

For peer review only - http://bmjopen.bmj.com/site/about/guidelines.xhtml

adherence in PWTB especially where DOTs programs are not designed and implemented appropriately [4-6]. The effectiveness of DOTs is influenced by higher DOTs coverage where cure rates have been found to be higher amongst those DOTs supporters with fewer patients allocated to them [5]. Digital adherence technology (DAT) including medication monitors may overcome challenges to monitoring tuberculosis treatment adherence through remotely documenting dosing patterns of PWTB [7].

Both globally and in South Africa, there is limited information on the value of DATs in TB treatment. A study done in KwaZulu-Natal SA amongst drug resistant TB-HIV co-infected inpatients using an older version of the Wisepill device, the RT2000 3G, showed that feasibility challenges for digital pillboxes may include battery failure, device malfunction and problems related to cellular networks [8]. Previous studies have found that other DAT such as SMS reminders for TB treatment were insufficient to improve treatment adherence alone. Barriers included frequent changing of phone numbers and uncertainty whether patients were taking their tablets immediately after they received the SMS or not [9]. Another study done in Uganda for HIV treatment showed that challenges for cellphone-based strategies included: use of shared cell phones, technical failures preventing receipt of SMS texts, electricity outages and changing phone numbers [10, 11]. Another DAT known as 99 DOTs also has the potential to improve treatment adherence as the PWTB is required to call a hidden phone number within the blister pack so as to indicate when a dose was taken [10]. However, 99 DOTS is limited in its ability to accurately report if a dose was taken since the PWTB may dial the number without actually taking any medication [10, 12]. This challenge has been overcome by DATs methods like Video DOT (VOT) which allow HCWs to watch the PWTB taking treatment over video-conferencing hence it is considered a more accurate method to ensure that PWTB adhere to treatment [13]. In addition, VOT can be a source of support to those who lack family and friends to support them during their TB illness [14]. However, VOT requires a smartphone, data plan and sufficient bandwidth making it a less affordable method in low and middle income countries such as SA [13].

For peer review only - http://bmjopen.bmj.com/site/about/guidelines.xhtml

Most DATs have focused on monitoring adherence and few studies have used DATs to differentiate between those patients who are struggling and need individual support, and those who are doing well. Given this gap in knowledge, we evaluated the feasibility of using the Wisepill evriMED DAT to monitor TB treatment adherence while using a differentiated care approach (DCA) with a stepwise increase in adherence support for PWTB in South Africa.

METHODS

Study design and study setting

This qualitative study was embedded within a cluster-randomized trial (CRT) that took place in six clinics in each of three provinces of South Africa: Gauteng (Ekurhuleni district); Western Cape (Klipfontein and Mitchell's Plain districts); and Kwa-Zulu Natal (eThekwini district), the details of which have been published elsewhere [15]. In the intervention arm of the CRT, PWTB received medication monitors with reminders triggering differentiated care approach (DCA) in response to adherence data uploads, carried out from a central database [15]. The DCA was implemented in a progressive manner depending on the number of doses a participant missed [15]. If one dose was missed, then a short message service (SMS) reminder was sent to the participant [15]. If a second or third dose was missed, then study staff would make a telephone call to the participant and once the fourth dose was missed then a home visit was conducted during which motivational counselling took place [15]. We describe the *feasibility* of implementing Wisepill evriMED device and differentiated care from a stakeholder perspective.

Site selection

There were a total of nine intervention clinics, three in each province. Clinics were selected based on location, HIV prevalence and numbers of patients starting TB treatment per month [15].

For peer review only - http://bmjopen.bmj.com/site/about/guidelines.xhtml

Study population

The study population consisted of seven purposively selected stakeholders from the Department of Health (DOH): one national, one provincial and five district-level representatives, who worked closely with the facilities using the electronic device (Wisepill evriMED DAT). From each intervention facility, we interviewed one facility staff (government employee) and one study staff member. All study staff worked on the project for at least three months. Facility staff-initiated patients on TB treatment and monitored patients for their scheduled monthly follow-up visits while study staff offered PWTB the device, followed-up on those who had missed doses and provided motivational and adherence counselling.

Patient and public involvement

Patients or the public were not involved in the design, or conduct, or reporting, or dissemination plans of our research.

Conceptual framework and themes explored

Using the feasibility framework suggested by Bowen et al [16], we developed an in depth interview (IDI) guide that covered the following topics; (i) the relative ease of implementation and operation of the technology within existing health systems, technology infrastructure and supply chain; (ii) system level challenges of delivering, sustaining and integrating the intervention into the existing TB programme. We used the framework on the 'Integration and sustainability of interventions into health systems' [17] to organize the data.

Data collection

We conducted a total of 25 in-depth interviews. The sample was representative of providers involved in the intervention while also considering saturation where themes usually start to converge after 15 interviews. The IDI guide was piloted with one stakeholder, two facility and

For peer review only - http://bmjopen.bmj.com/site/about/guidelines.xhtml

one study staff between January to February 2020. The probing questions were adapted after the pilot. These interviews were conducted face-to-face, and the data was included in the analysis. Data collection continued between June 2020 to January 2021 with each interview lasting between 45 – 90 minutes. The interviews were all conducted in the providers preferred language by a female PhD student (RM) and a female study coordinator who was a PhD student with qualitative experience (VM). Both researchers established a prior relationship with the providers enabling them to understand the reasons for the study. The interviews were digitally recorded with the providers consent and transcribed verbatim by trained research assistants (Masters students). The transcripts were not returned to the providers for comment or correction. Due to the impact of COVID-19 and the national lockdown that took place, these interviews were conducted virtually over Microsoft Teams. No one else was present in the interviews besides the providers and the researchers and field notes were made during the interviews. Saturation was assessed during data collection through asking the same question in different ways and reviewing a sample of the recordings until no new information was obtainable. No repeat interviews were carried out.

Data analysis

Thematic analysis was used with deductive and inductive approaches [18, 19]. At least 10% of the transcripts were coded inductively by two independent researchers so as to reduce bias and improve reliability [18]. A codebook of emerging themes was developed, guided by the framework on the 'Integration and sustainability of interventions into health systems' [17],with discrepancies being resolved through discussion. Where there was no inter-rater agreement, the theme was dropped [18]. The final codebook was used to code the remaining transcripts and any new codes that emerged were also included in this codebook. MAXQDA qualitative software was used for the coding process. We highlight the major themes (overarching theme) and sub-themes (specific themes) that emerged and use supportive direct quotations from providers. Providers did not provide feedback on the findings.

For peer review only - http://bmjopen.bmj.com/site/about/guidelines.xhtml

RESULTS

We conducted 25 interviews in total with 18 staff (9 facility staff and 9 study staff) and 7 stakeholders (1 national,1 provincial and 5 at district-level) across 3 provinces. Majority of the providers (23 out of 25) were female. None of the approached providers declined to participate. Major themes included (1) Providers were supportive of the intervention, (2) Intervention challenges within the adoption system and broad context, and (3) Ensuring intervention sustainability through constant training of staff and education of PWTB

Major Theme 1: Providers were supportive of the intervention

There was buy-in from staff who felt involved and were supportive of the intervention. Stakeholders were also very supportive of the intervention despite their limited involvement.

> *"… to me it was a very good idea and it was working very well … it improved our cure rate because we had a lot of patients who were defaulting and who and to be referred to the hospital because they developed multi, MDR. So since we had …this intervention, at least we had less patients who developed multi-drug resistance." - FFS_019_7*

> *"…I think…it's magnificent. I think it's… a tool box, it's really a way for us to see what's really happening at the point of … TB treatment, where the patient take… the medication when they open the box. So I think it's really innovation that can be used. ... I think it also is a reminder for the patient. You know? That … "I need to take my medication". And it's a way to …to ensure the quality of the programme, that patients are adhering to the ... medication…I think it is a critical intervention." – FDS_WC_008 (District level)*

Both facility and study staff found the device easy to learn and facility staff who had received a briefing on the study were eager to receive the complete training to support the study staff.

For peer review only - http://bmjopen.bmj.com/site/about/guidelines.xhtml

"Because sometimes she (referring to study staff) [might not be here and it happens that she's not here, either she is sick or she's gone on holiday for December. She says: "…we can show you and then you do this things when we are not here". I said: "… it will be easy… As long as you show us, we can do that device".
- FFS_762_003

The staff described the activities of the intervention such as issuing devices, phone calls and making home visits as being well integrated into the TB programme. Providers had strong support for the device for two main reasons that we classified as sub-themes in our analysis:

Sub-theme 1: Device as a useful reminder and early notification tool

Most providers found the alarm that was fitted on the device to be a useful reminder to patients to take their treatment on time and to attend their clinic visits. The device also alerted providers to PWTB who were not using the device and not taking treatment.

"I feel like it assists with the real time monitoring because now if you check on the system, you would be able to check instead of waiting like someone waiting for their appointment that is months after, you can actually check now on the system that this person there's no activity, let me call and you would actually find that the person has died or maybe the person has- is in hospital is admitted.."- FSS_762_006

"So, it will help us because …sometimes we notice the other patient…didn't take their medication and start to recall all of them and then the other just default … With the box, someone is getting … a notification that this person is not taking her treatment..." - FFS_524_006

However, some staff distrusted that opening of the device meant that PWTB took their pills.

For peer review only - http://bmjopen.bmj.com/site/about/guidelines.xhtml

"…some patients do that when they open the pill box and … show an adherence of 100%, but still they… have not been taking their medications. So we would try to motivate them, counsel them, explain the disadvantages of not of them not taking the medication." - FSS_519_001

"I think because you know patients always find a way to find loopholes within the health system, so at some point they will understand that you're recording the opening and the closing of the box not necessarily them actively taking the medications. So they can easily open and close the box without really taking the medication." - FDS_GP_008 (District level)

Sub-theme 2: Differentiated Care Approach allows for patient-centred care

Staff mentioned that they found DCA to be a positive and unique aspect of our intervention.

"So, you are no longer administering intervention for one. You sort of trying to be specific …to a person and offer them care in their in their specific sort of situations. So, I think that is a positive thing because you don't assume everyone is the same. You don't assume everyone's situation is the same. You understand that you are dealing with … individuals. That's … what I think is positive about this differentiated model of care." - MSS_732_005

"… yah, I think I think the differentiated model of care … is a very positive thing, that it works to an extent because it's not a blanket approach. You don't, you don't think of every patient as the same…you sort of attend to each person in their context and … try to understand what they are going through." - MSS_732_005

For peer review only - http://bmjopen.bmj.com/site/about/guidelines.xhtml

The staff also felt that the counselling they provided as part of the DCA helped them to understand their patients' reasons for not taking their treatment thus allowing them to manage them better as they supported them on their treatment journey.

"This whole approach I feel like it's a great initiative because it assists in managing the patient - like fully. Like if I can say wholly, not just managing the patient, taking the medication. You could also like… with the counselling you actually find that the problem is that the participant is facing beyond treatment intake."- FSS_762_006

Phone calls to PWTB allowed for clarification on any issues that may not have been clear to the patient while at the clinic.

"…he didn't understand but when I called him and I was speaking to him over the phone to understand how they're taking the treatment, I find that … yeah there was mistake … one of the best things about this intervention because some of them are able to even call me up, I don't always now have to call patients."

- MSS_732_005

Home visits allowed the staff to have better interaction with their patients and to build trust with them making them feel like someone cares about them.

"It's not bad. It's nice to do home visits. It's whereby … you contact with the patients, know each other … where the patient has got the problem, it's with the … problem with the box or she wanted to ask me something that she forget to ask at the clinic."

- FSS_522_002

"… I think the participant at least get that thing when you do home visits, or you phone them when they are not taking the treatment they feel as if they are cared of,

For peer review only - http://bmjopen.bmj.com/site/about/guidelines.xhtml

someone is care is cared for me when I am not taking the treatment because I would get a call, or I will get a home visit''- FSS_524_003

Some providers described DCA as more of a journey of supporting the patients and helping build trust between the staff and the PWTB.

"… that is really good part of the intervention. I think it … continue to build that trust…And that support for the patients … so I- I think that was impressive." - FDS_WC_008 (District level)

"I thought it was only about the box… it's more than them just using the box. It's their treatment journey and being supported to adhere to the treatment and get healed from the TB." - FSS_762_006

Major Theme 2: Intervention challenges within the adoption system and broad context of South Africa

Some staff felt that the trust they built with the PWTB could be threatened by human resource shortages and a dedicated cadre was needed to ensure successful implementation.

"However, when we find at once the research study stop and we over onto implementation, … the constraints is always HR resources and at the operational level, if that initial education isn't framed correctly. There could be a misunderstanding about the purpose of the box." - FDS_WCR_008 (District level)

Challenges that were encountered with the device or the network resulted in delays in the system updating that the box was opened which staff felt created trust issues between them and the participant.

For peer review only - http://bmjopen.bmj.com/site/about/guidelines.xhtml

“Now it’s creating a trust issue between you and the patient, because you phoning regarding treatment that was not taken because … Wisepill says treatment was not taken, while the patient on the other side did take the medication.”- FSS_516_002

Heath care workers reported that some patients received incorrect SMS’s due to delays in the system.

‘’ Especially when it has been a weekend and then they will not … send out the data of… the weekend like … if there were any missed or any … intakes. Sometimes they will show that they missed the weekend and they did not take the medication, but in three days down the line… the correct information will show. That’s where we will see that there were no missed doses.’’- FSS_519_001

Staff also felt that that the device would not work for some group of patients such as those who abuse substances.

“The patient that are on substance abuse, yoh! You know, when they come here, they are ok. At a later stage, once they’ve started treatment, you realise that this patient is using substances…With those patients, they the device- ya. It’s not good. Won’t- won’t work. Won’t- won’t help.” - FFS_762_003

“He took the evriMED box device and the TB meds. That was the last time we saw him on the clinic. He run away from home apparently. He was on drugs. But then…he is back. He’s here in the TB room. He’s got TB again. So I was asking him the other day: “are you still interested in joining this study?”. And he was like: “you know what I did with the first box? I sold it”. - FSS_521_001

For peer review only
For peer review only - http://bmjopen.bmj.com/site/about/guidelines.xhtml

Sub-theme 1: Provider perceptions of stigma related to use of the evriMED device and DCA

Staff perceived that some of the features of the box such as the alarm and the size of the box may have been a concern to some PWTB in trying to conceal their TB status so they would opt to leave their devices behind when travelling on holiday or going to work for fear of disclosure of their TB status.

> *"The thing that is common, especially in December … an example is person supposed to take it at 8, they take it at 8 but they not taking it at 8 from the box... I think they went for holidays … This person is taking treatment and the putting it… in a purse. Whereby you'll take in the morning without this alarm ringing.''- MSS_019_002*

> *"So some will come to us and say: "what if the pill box doesn't make so much noise? It will be easier for me to carry around- or if it was a bit … smaller, then it will be easier for me to carry it. But now it's big, I don't want … people seeing me like with this pill box in the taxi or at work."- FSS_519_001*

> *"I haven't disclosed to my partner that I'm on TB medication and imagine if I had to carry this box and the box would be ringing in the morning and it would cause unnecessary fights." - FSS_762_006*

Providers perceived that stigma was more of an issue outside the PWTB household as often they would not disclose their TB status to members outside their homes.

> *"So, I noticed that it's comfortable for them to use this box when they are at home with the people that actually know what is going on with them but when they are*

For peer review only

going to other people that are not aware that they are sick or anything, it seems as if they don't want to carry the boxes." - FSS_762_006

Staff perceived that stigma related to DCA existed in the community and some of them witnessed the PWTB fear of being stigmatized when conducting home visits. PWTB would often ask staff not to wear their uniforms when they visit their homes.

"Uhm they hate it when we wear our TB MATE t-shirts, because they are saying: "due to stigma". When we go visit their homes, we must not wear something that will be written 'TB' or 'HIV'. So we wear our normal clothes..."- FSS_521_001

"Then I took off my jacket, and then the participant saw that TB MATE on my t-shirt. And then the patient told me that I must wear my jacket because what if someone walks in and sees the TB MATE on my t-shirt. So I wasn't aware that the participant didn't disclose... She told me that she's got fear that- because she will be judged because she was drinking alcohol a lot. But now she's got TB. They will say she must go and stay in the back room."- FSS_521_001

Staff perceived that some stigma could have been internalised and not actually experienced by PWTB.

"It's their thoughts because the person would feel like it did not want to be seen carrying the box. Because people will think- so he sees all their thoughts. It's not something that really happened."- FFS_732_001

"But in the clinic, there wasn't really anything that contributed to the stigma but maybe with now giving the pill box… some of the patients would say that maybe it's gonna show. Maybe with their ARV's they could take it in private. Now we have them

For peer review only - http://bmjopen.bmj.com/site/about/guidelines.xhtml

having this pill box that is gonna ring even, that is gonna beep and remind them to take treatment...'' - FSS_732_005

Major Theme 3: Ensuring intervention sustainability through constant training of staff and education of PWTB

Education on the intervention emerged as one of the major themes that stakeholders and staff placed emphasis on as a means of managing their patients in a holistic way leading to successful treatment outcomes. Education and training should emphasize the importance of adhering to treatment and should be strengthened at various levels – with the PWTB themselves, those who would be involved in implementation such as community health workers (CHWs) and also to the community at large.

> *''My experience was that as the community we need to need to educate the community the importance of complying to medication…we need to educate and educate and educate.''- FFS_019_007*

> *"But I definitely think it's… strengthening that interface between the patient and the staff... it's an important interface. And- and of course that too- the 'how' of- of the contact, if it is about 'why you didn't- why you aren't taking your treatment' rather than saying 'how are you doing'- you know- 'are you ok', uhm 'how can I help you'. When we start off with those different conversations, it can lead to different outcomes." - FDS_WC_008 (District level)*

The staff felt that constant education and reinforcement would ultimately lead to behaviour change and treatment adherence.

> *"I think the intervention even though we are the one offering the intervention but we sort of gave patients the power to understand the… reasons why they are taking*

For peer review only - http://bmjopen.bmj.com/site/about/guidelines.xhtml

treatment, why is it important for them to…adhere to the treatment…we were actually able to make patients to take their treatment journey into their own hands … it's almost like we were empowering patients…"- MSS_732_005

DISCUSSION

Using the Wisepill evriMED device to support adherence to TB treatment appears to be a feasible option in South Africa. Providers were supportive of the evriMED device and the differentiated care approach, as they felt it had a positive impact on the TB patient's programme. Stakeholders were supportive despite their involvement being limited to approval of the study and providing oversight of the TB programme.

This study has shown that DATs is an innovative approach that might improve the management of PWTB by allowing individual differentiated support. Early notification of missed doses from the device allowed staff to intervene through SMS messaging and phone calls, thus reducing the need for a home visit. This freed up time for staff to focus on other duties. These findings are similar to other studies where differentiated care models used in HIV treatment delivery have addressed challenges such as overcrowded facilities, overburdened staff and long waiting times in countries like Uganda, Swaziland, Mozambique and South Africa [7, 20-22].

Staff noted that the differentiated care allowed a journey of educating and supporting PWTB. Through adherence and motivational counselling, staff empowered patients to understand the importance of adhering to treatment and to empower them to take their treatment journey into their own hands. For scale-up, the real time monitoring feature of the device is important so that the correct individuals are promptly identified for further support before they become lost to follow-up. This also allows for the efficient utilisation of resources to those in most need, as seen in various studies [6, 23, 24]. More attention should also be given to

For peer review only - http://bmjopen.bmj.com/site/about/guidelines.xhtml

relationship training as the sustainability of the intervention depends on a sound provider-patient relationship. Correct framing of the intervention as a support tool would also help ensure that the intervention is well perceived once scaled-up. Training needs to be constant and not once-off to reinforce their understanding of the intervention. In a routine TB program setting, training of healthcare providers could be conducted by sub-district co-ordinators since they visit facilities on a regular basis for monitoring purposes.

Some challenges with the technology were cited such as alarm malfunction, incorrectly sent SMS's and short battery lifespan. These challenges created distrust from PWTB, and the device would therefore need to be of the highest quality to ensure sustainability of the intervention. A systematic review of DAT for the management of tuberculosis therapy found similar feasibility challenges remain in low- and middle-income settings [10].Once the intervention is scaled up, the shortage of human resources (HR) could serve as a barrier to ensuring that providers receive the correct information. For successful scale-up, some staff recommended that a dedicated cadre should be in place to follow-up PWTB to ensure that they understand the importance of taking treatment. However, since the support system had reduced the need for home visits and follow up calls, the current TB cadres, clerical staff and community-based teams were also seen as being sufficient. Despite any challenges experienced within the adoption system and broad context, the staff were all very supportive of the intervention and even recommended that it should be used for all TB and chronic patients as well as in other facilities.

Fear of stigma and the disclosure of one's TB status has the potential to act as a barrier to scale-up and sustainability of the intervention, if not well addressed. Stigma and fear of disclosure resulting in lower acceptance of DAT technologies amongst multi-drug resistant TB patients have also been reported in India [25], with improvements recommended on the design. Disease-related stigma may be more difficult to address, and screening should be

For peer review only

used to identify upfront those patients whom stigma and fear of disclosure may limit the use of the MERM [25].

A limitation of the study was that interviews had to be conducted virtually on Microsoft teams, due to the Covid-19 pandemic, and to allow for effective social distancing for both the researchers and the study providers. This new mode of conducting interviews had setbacks such as network and connectivity challenges which interrupted the flow of some of the interviews. The interviewers mitigated this challenge through re-iterating what the participant had said to ensure that the correct message had been captured. Through the consenting process, we were able to ensure confidentiality and that the participant was relaxed. A second limitation is that some of the interviews were conducted by a member of the study management team, although this was limited to senior stakeholders who were unlikely to feel reluctant to freely express their views and to withhold some of their opinions. Patients were not included in this study which is a limitation since their views would have added a broader understanding on feasibility. We conducted the study in three provinces with different health service characteristics, TB epidemiology and population characteristics which allowed us to understand feasibility of implementation from a broader perspective. Another strength was that the study was done in a routine setting with limited resources and without the use of incentives to providers to increase uptake.

CONCLUSION

Digital adherence technology (DAT) such as medication monitors have a huge potential to improve tuberculosis treatment adherence. However, to ensure successful scale-up and sustainability of the intervention, the differentiated care approach should be used as a platform to constantly educate PWTB and the community at large on TB and the importance of adhering to treatment since technology on its own will not solve treatment adherence issues especially those related to stigma and lack of support. Sound communication

For peer review only - http://bmjopen.bmj.com/site/about/guidelines.xhtml

between PWTB and providers serves as a key tool to improving treatment outcomes thus more attention should be given to relationship training to ensure that the provider-patient relationships provide the necessary support to those who need it most.

ABBREVIATIONS

COVID	Corona Virus Disease
DCA	Differentiated Care Approach
DOH	Department of Health
DS-TB	Drug-sensitive TB
DATs	Digital Adherence Technologies
DOTs	Directly Observed Treatment Short Course
ICFs	Informed Consent Forms
IDI	In-depth Interview
MDR-TB	Multidrug-resistant TB
PWTB	People With TB
SA	South Africa
SMS	Short Message Service
TB	Tuberculosis
TB MATE	TB Monitoring Adherence to Treatment Endpoints

ACKNOWLEDGEMENTS

We would like to thank the following: Ekurhuleni, City of Cape Town, eThekwini districts and the Ekurhuleni Health District Research Committee (EHDRC) for allowing us to conduct the study in their districts. The study coordinators from Gauteng, Western Cape and KwaZulu-Natal in South Africa for their assistance with data collection namely Israel Rabothata, Vuyelwa Mehlomakulu (VM), Vumile Gumede and Bongani Zondi and their field-based teams of research assistants and interns; all the transcribers who dedicated time to capture the conversations verbatim; all the providers for taking part in this study

CONTRIBUTORSHIP STATEMENT

RM, NM, LJ, MTM, KLF, SC and CMCM conceptualized the study. RM and CMCM analysed the data. RM, NM, CO, SC and CMCM interpreted the data. RM wrote the original draft and revised subsequent versions of the manuscript. SC and CMCM provided guidance on earlier versions of the manuscript. NM, CO, LJ, PN, MTM, KV, KLF, SC and CMCM reviewed and edited previous versions. RM, SC and CMCM are responsible for the overall content of the manuscript. The authors read and approved the final manuscript.

COMPETING INTERESTS

The authors declare that they have no competing interests.

For peer review only

FUNDING STATEMENT

The study is funded by (1) Bill & Melinda Gates Foundation (OPP1205388), (2) TB REACH Wave 6 project of Stop TB Partnership (STBP/TBREACH/GSA/W6-34), and (3) South African Medical Research Council through the Strategic Health Innovation Partnerships. Funders did not play a part in the design of the study or the decision to submit the manuscript for publication.

DATA SHARING STATEMENT

No additional data available.

ETHICS APPROVAL AND PARTICIPANT CONSENT

The parent study received ethics approval from the three district and provincial ethics committees where the trial sites were located. This qualitative study was approved by the Human Research Ethics Committee (HREC) of the University of Witwatersrand (Ref 180705) and the University of Cape Town (Ref 452/2018). We obtained written informed consent for study participation and permission for digital recording from all providers. Providers were not reimbursed.

REFERENCES

1. WHO. Global Tuberculosis Report 2021. Geneva: World Health Organization 2021 [Available from: https://worldhealthorg.shinyapps.io/tb_profiles/?_inputs_&entity_type=%22country%22&lan=%22EN%22&iso2=%22ZA%22.
2. WHO. Global Tuberculosis Report 2018. Geneva: World Health Organization.
3. Cramm JM, Finkenflugel HJ, Moller V, Nieboer AP. TB treatment initiation and adherence in a South African community influenced more by perceptions than by knowledge of tuberculosis. BMC Public Health. 2010;10:72.
4. Knight M, van Zyl RL, Sanne I, Bassett J, van Rie A. Impact of combination antiretroviral therapy initiation on adherence to antituberculosis treatment. South Afr J HIV Med. 2015;16(1):346.
5. Ntshanga SP, Rustomjee R, Mabaso ML. Evaluation of directly observed therapy for tuberculosis in KwaZulu-Natal, South Africa. Trans R Soc Trop Med Hyg. 2009;103(6):571-4.
6. Serapelwane MG, Davhana-Maselesele M, Masilo GM. Experiences of patients having tuberculosis (TB) regarding the use of Directly Observed Treatment Short-Course (DOTS) in the North West Province, South Africa. Curationis. 2016;39(1):e1-e9.
7. Liu X, Lewis JJ, Zhang H, Lu W, Zhang S, Zheng G, et al. Effectiveness of Electronic Reminders to Improve Medication Adherence in Tuberculosis Patients: A Cluster-Randomised Trial. PLoS Med. 2015;12(9):e1001876.
8. Bionghi N, Daftary A, Maharaj B, Msibi Z, Amico KR, Friedland G, et al. Pilot evaluation of a second-generation electronic pill box for adherence to Bedaquiline and antiretroviral therapy in drug-resistant TB/HIV co-infected patients in KwaZulu-Natal, South Africa. BMC Infect Dis. 2018;18(1):171.
9. Hüsler J. Evaluation of the On Cue Compliance Service Pilot : Testing the use of SMS reminders in the treatment of Tuberculosis in Cape Town, South Africa. 2005.
10. Subbaraman R, de Mondesert L, Musiimenta A, Pai M, Mayer KH, Thomas BE, et al. Digital adherence technologies for the management of tuberculosis therapy: mapping the landscape and research priorities. BMJ Glob Health. 2018;3(5):e001018.

For peer review only - http://bmjopen.bmj.com/site/about/guidelines.xhtml

11. Hermans SM, Elbireer S, Tibakabikoba H, Hoefman BJ, Manabe YC. Text messaging to decrease tuberculosis treatment attrition in TB-HIV coinfection in Uganda. Patient Prefer Adherence. 2017;11:1479-87.
12. Cross A, Gupta N, Liu B, Nair V, Kumar A, Kuttan R, et al. 99DOTS: A Low-Cost Approach to Monitoring and Improving Medication Adherence. Tenth International Conference on Information and Communication Technologies and Development (ICTD '19). 2019.
13. Story A, Aldridge RW, Smith CM, Garber E, Hall J, Ferenando G, et al. Smartphone-enabled video-observed versus directly observed treatment for tuberculosis: a multicentre, analyst-blinded, randomised, controlled superiority trial. Lancet. 2019;393(10177):1216-24.
14. Fraser H, Keshavjee S. Video-observed therapy for tuberculosis: strengthening care. Lancet. 2019;393(10177):1180-1.
15. Maraba N, Orrell C, Chetty-Makkan CM, Velen K, Mukora R, Page-Shipp L, et al. Evaluation of adherence monitoring system using evriMED with a differentiated response compared to standard of care among drug-sensitive TB patients in three provinces in South Africa: a protocol for a cluster randomised control trial. Trials. 2021;22(1):389.
16. Bowen DJ, Kreuter M, Spring B, Cofta-Woerpel L, Linnan L, Weiner D, et al. How we design feasibility studies. Am J Prev Med. 2009;36(5):452-7.
17. Atun R, de Jongh T, Secci F, Ohiri K, Adeyi O. Integration of targeted health interventions into health systems: a conceptual framework for analysis. Health Policy Plan. 2010;25(2):104-11.
18. Pope C, Ziebland S, Mays N. Qualitative research in health care. Analysing qualitative data. BMJ. 2000;320(7227):114-6.
19. Charmaz K. Constructing Grounded Theory: A practical guide through qualitative analysis Nurse Res. 2006;13(4):84.
20. Wilkinson L, Harley B, Sharp J, Solomon S, Jacobs S, Cragg C, et al. Expansion of the Adherence Club model for stable antiretroviral therapy patients in the Cape Metro, South Africa 2011-2015. Trop Med Int Health. 2016;21(6):743-9.
21. Decroo T, Telfer B, Biot M, Maikere J, Dezembro S, Cumba LI, et al. Distribution of antiretroviral treatment through self-forming groups of patients in Tete Province, Mozambique. J Acquir Immune Defic Syndr. 2011;56(2):e39-44.
22. Okoboi S, Ding E, Persuad S, Wangisi J, Birungi J, Shurgold S, et al. Community-based ART distribution system can effectively facilitate long-term program retention and low-rates of death and virologic failure in rural Uganda. AIDS Res Ther. 2015;12:37.
23. Kurz SG, Furin JJ, Bark CM. Drug-Resistant Tuberculosis: Challenges and Progress. Infect Dis Clin North Am. 2016;30(2):509-22.
24. Subbaraman R, Nathavitharana RR, Mayer KH, Satyanarayana S, Chadha VK, Arinaminpathy N, et al. Constructing care cascades for active tuberculosis: A strategy for program monitoring and identifying gaps in quality of care. PLoS Med. 2019;16(2):e1002754.
25. Thomas BE, Kumar JV, Periyasamy M, Khandewale AS, Hephzibah Mercy J, Raj EM, et al. Acceptability of the Medication Event Reminder Monitor for Promoting Adherence to Multidrug-Resistant Tuberculosis Therapy in Two Indian Cities: Qualitative Study of Patients and Health Care Providers. J Med Internet Res. 2021;23(6):e23294.

For peer review only - http://bmjopen.bmj.com/site/about/guidelines.xhtml

COREQ (COnsolidated criteria for REporting Qualitative research) Checklist

A checklist of items that should be included in reports of qualitative research. You must report the page number in your manuscript where you consider each of the items listed in this checklist. If you have not included this information, either revise your manuscript accordingly before submitting or note N/A.

Topic	Item No.	Guide Questions/Description	Reported on Page No.
**Domain 1: Research team and reflexivity**			
Personal characteristics			
Interviewer/facilitator	1	Which author/s conducted the interview or focus group?	
Credentials	2	What were the researcher's credentials? E.g. PhD, MD	
Occupation	3	What was their occupation at the time of the study?	
Gender	4	Was the researcher male or female?	
Experience and training	5	What experience or training did the researcher have?	
Relationship with participants			
Relationship established	6	Was a relationship established prior to study commencement?	
Participant knowledge of the interviewer	7	What did the participants know about the researcher? e.g. personal goals, reasons for doing the research	
Interviewer characteristics	8	What characteristics were reported about the inter viewer/facilitator? e.g. Bias, assumptions, reasons and interests in the research topic	
**Domain 2: Study design**			
Theoretical framework			
Methodological orientation and Theory	9	What methodological orientation was stated to underpin the study? e.g. grounded theory, discourse analysis, ethnography, phenomenology, content analysis	
Participant selection			
Sampling	10	How were participants selected? e.g. purposive, convenience, consecutive, snowball	
Method of approach	11	How were participants approached? e.g. face-to-face, telephone, mail, email	
Sample size	12	How many participants were in the study?	
Non-participation	13	How many people refused to participate or dropped out? Reasons?	
Setting			
Setting of data collection	14	Where was the data collected? e.g. home, clinic, workplace	
Presence of non-participants	15	Was anyone else present besides the participants and researchers?	
Description of sample	16	What are the important characteristics of the sample? e.g. demographic data, date	
Data collection			
Interview guide	17	Were questions, prompts, guides provided by the authors? Was it pilot tested?	
Repeat interviews	18	Were repeat inter views carried out? If yes, how many?	
Audio/visual recording	19	Did the research use audio or visual recording to collect the data?	
Field notes	20	Were field notes made during and/or after the inter view or focus group?	
Duration	21	What was the duration of the inter views or focus group?	
Data saturation	22	Was data saturation discussed?	
Transcripts returned	23	Were transcripts returned to participants for comment and/or	

For peer review only

Topic	Item No.	Guide Questions/Description	Reported on Page No.
		correction?	
**Domain 3: analysis and findings**			
Data analysis			
Number of data coders	24	How many data coders coded the data?	
Description of the coding tree	25	Did authors provide a description of the coding tree?	
Derivation of themes	26	Were themes identified in advance or derived from the data?	
Software	27	What software, if applicable, was used to manage the data?	
Participant checking	28	Did participants provide feedback on the findings?	
Reporting			
Quotations presented	29	Were participant quotations presented to illustrate the themes/findings? Was each quotation identified? e.g. participant number	
Data and findings consistent	30	Was there consistency between the data presented and the findings?	
Clarity of major themes	31	Were major themes clearly presented in the findings?	
Clarity of minor themes	32	Is there a description of diverse cases or discussion of minor themes?	

Developed from: Tong A, Sainsbury P, Craig J. Consolidated criteria for reporting qualitative research (COREQ): a 32-item checklist for interviews and focus groups. *International Journal for Quality in Health Care*. 2007. Volume 19, Number 6: pp. 349 – 357

Once you have completed this checklist, please save a copy and upload it as part of your submission. DO NOT include this checklist as part of the main manuscript document. It must be uploaded as a separate file.

For peer review only

BMJ Open

A qualitative study exploring the feasibility of using medication monitors and a differentiated care approach to support adherence among people receiving TB treatment in South Africa

Journal:	*BMJ Open*
Manuscript ID	bmjopen-2022-065202.R2
Article Type:	Original research
Date Submitted by the Author:	14-Feb-2023
Complete List of Authors:	Mukora, Rachel; The Aurum Institute<br>Maraba, Noriah; The Aurum Institute<br>Orrell, Catherine; University of Cape Town Department of Medicine, Institute of Infectious Disease and Molecular Medicine; Desmond Tutu HIV Foundation<br>Jennings , Lauren; Desmond Tutu HIV Foundation<br>Naidoo, P.; University of Stellenbosch<br>Mbatha, M.; Interactive Research and Development<br>Velen, Kavindhran ; The Aurum Institute<br>Fielding, Katherine; London School of Hygiene & Tropical Medicine, TB Centre; University of the Witwatersrand School of Public Health<br>Charalambous, Salome; The Aurum Institute; University of the Witwatersrand School of Public Health<br>Chetty-Makkan, Candice; Health Economics and Epidemiology Research Office
**Primary Subject Heading**:	Qualitative research
Secondary Subject Heading:	Public health, Global health
Keywords:	Public health < INFECTIOUS DISEASES, Tuberculosis < INFECTIOUS DISEASES, Health policy < HEALTH SERVICES ADMINISTRATION & MANAGEMENT, Rationing < HEALTH SERVICES ADMINISTRATION & MANAGEMENT, Organisation of health services < HEALTH SERVICES ADMINISTRATION & MANAGEMENT

SCHOLARONE™
Manuscripts

I, the Submitting Author has the right to grant and does grant on behalf of all authors of the Work (as defined in the below author licence), an exclusive licence and/or a non-exclusive licence for contributions from authors who are: i) UK Crown employees; ii) where BMJ has agreed a CC-BY licence shall apply, and/or iii) in accordance with the terms applicable for US Federal Government officers or employees acting as part of their official duties; on a worldwide, perpetual, irrevocable, royalty-free basis to BMJ Publishing Group Ltd ("BMJ") its licensees and where the relevant Journal is co-owned by BMJ to the co-owners of the Journal, to publish the Work in this journal and any other BMJ products and to exploit all rights, as set out in our licence.

The Submitting Author accepts and understands that any supply made under these terms is made by BMJ to the Submitting Author unless you are acting as an employee on behalf of your employer or a postgraduate student of an affiliated institution which is paying any applicable article publishing charge ("APC") for Open Access articles. Where the Submitting Author wishes to make the Work available on an Open Access basis (and intends to pay the relevant APC), the terms of reuse of such Open Access shall be governed by a Creative Commons licence – details of these licences and which Creative Commons licence will apply to this Work are set out in our licence referred to above.

Other than as permitted in any relevant BMJ Author's Self Archiving Policies, I confirm this Work has not been accepted for publication elsewhere, is not being considered for publication elsewhere and does not duplicate material already published. I confirm all authors consent to publication of this Work and authorise the granting of this licence.

For peer review only

Title: A qualitative study exploring the feasibility of using medication monitors and a differentiated care approach to support adherence among people receiving TB treatment in South Africa

Authors: Rachel Mukora[1,8§], Noriah Maraba[1], Catherine Orrell[2,3], Lauren Jennings[3], Pren Naidoo[4], M.Thulani Mbatha[5], Kavindhran Velen[1], Katherine L.Fielding[6,8], Salome Charalambous[1,8], Candice M Chetty-Makkan[7]

Affiliations

1. The Aurum Institute, Aurum House, Parktown, Johannesburg, South Africa
2. University of Cape Town, Department of Medicine, and Institute of Infectious Disease and Molecular Medicine, Cape Town, South Africa
3. Desmond Tutu Health Foundation, Cape Town, South Africa
4. Stellenbosch University, Stellenbosch, South Africa
5. Interactive Research and Development, Durban, South Africa
6. London School of Hygiene and & Tropical Medicine, United Kingdom
7. Health Economics and Epidemiology Research Office, Faculty of Health Sciences, University of the Witwatersrand, Johannesburg, South Africa
8. University of Witwatersrand, School of Public Health, Johannesburg, South Africa

§Corresponding author

Name: Rachel Mukora

Address: The Aurum Institute, Aurum House, The Ridge, 29 Queens Road, Parktown, Johannesburg, 2193, South Africa

Email: rmukora@auruminstitute.org

Keywords

Digital Adherence Technology, Differentiated Care Approach, treatment adherence support, Tuberculosis, Feasibility

Word count abstract: 299

Word count manuscript: 4816

For peer review only - http://bmjopen.bmj.com/site/about/guidelines.xhtml

ABSTRACT

Objectives

The TB MATE study evaluated whether a differentiated care approach (DCA) based on tablet-taking data from Wisepill evriMED digital adherence technology could improve tuberculosis treatment adherence. The DCA entailed a stepwise increase in adherence support starting from short message service (SMS) to phone calls, followed by home visits and motivational counselling. We explored feasibility of this approach with providers in implementing clinics.

Design

Between June 2020 to February 2021, In-depth interviews were conducted in the provider's preferred language, audio recorded, transcribed verbatim and translated. The interview guide included three categories: feasibility, system-level challenges and sustainability of the intervention. We assessed saturation and used thematic analysis.

Setting

Primary healthcare clinics in three provinces of South Africa.

Participants

We conducted 25 interviews with 18 staff and seven stakeholders.

Results

Three major themes emerged: Firstly, providers were supportive of the intervention being integrated into the TB programme and were eager to be trained on the device as it helped to monitor treatment adherence. Secondly, there were challenges in the adoption system such as shortage of human resources which could serve as a barrier to information provision once the intervention is scaled-up. Heath care workers reported that some patients received incorrect SMS's due to delays in the system that contributed to distrust. Thirdly, DCA was considered as a key aspect of the intervention by some staff and stakeholders since it allowed for support based on individual needs.

For peer review only - http://bmjopen.bmj.com/site/about/guidelines.xhtml

Conclusions

It was feasible to monitor tuberculosis treatment adherence using the evriMED device and DCA. To ensure successful scale-up of the adherence support system, emphasis will need to be placed on ensuring that the device and the network operate optimally and continued support on adhering to treatment which will enable people with TB to take ownership of their treatment journey and help overcome TB related stigma.

Strengths and limitations of this study

- We conducted the study in three provinces with different health service characteristics, TB epidemiology and population characteristics which allowed us to understand feasibility of implementation from a broader perspective.
- Another strength was that the study was done in a routine setting with limited resources and without the use of incentives to providers to increase uptake.
- One limitation is that some of the interviews were conducted by a member of the study management team, although this was limited to senior stakeholders who were unlikely to feel reluctant to freely express their views and to withhold some of their opinions.

INTRODUCTION

Globally the treatment success rate for new and relapse drug-sensitive TB (DS-TB), is 86% while in South Africa (SA) this is much lower at 79% [1]. Amongst those who are HIV positive with TB, the treatment success rate is 78% and 67% for those previously treated with TB [1]. Treatment failure is often a result of non-adherence to treatment, loss to follow-up or unevaluated outcomes [2]. Typical reasons that people with TB (PWTB) might not adhere to treatment include false perceptions of being cured once they feel better, stigma, forgetfulness, lack of social network support, and poor user experience of accessing care at clinics [3]. Traditional methods such as Directly Observed Treatment Short course (DOTs), pill counts and self-report have limitations and have not been shown to improve treatment

For peer review only - http://bmjopen.bmj.com/site/about/guidelines.xhtml

adherence in PWTB especially where DOTs programs are not designed and implemented appropriately [4-6]. The effectiveness of DOTs is influenced by higher DOTs coverage where cure rates have been found to be higher amongst those DOTs supporters with fewer patients allocated to them [5]. Digital adherence technology (DAT) including medication monitors may overcome challenges to monitoring tuberculosis treatment adherence through remotely documenting dosing patterns of PWTB [7].

Both globally and in South Africa, there is limited information on the value of DATs in TB treatment. A study done in KwaZulu-Natal SA amongst drug resistant TB-HIV co-infected inpatients using an older version of the Wisepill device, the RT2000 3G, showed that feasibility challenges for digital pillboxes may include battery failure, device malfunction and problems related to cellular networks [8]. Previous studies have found that other DAT such as SMS reminders for TB treatment were insufficient to improve treatment adherence alone. Barriers included frequent changing of phone numbers and uncertainty whether patients were taking their tablets immediately after they received the SMS or not [9]. Another study done in Uganda for HIV treatment showed that challenges for cellphone-based strategies included: use of shared cell phones, technical failures preventing receipt of SMS texts, electricity outages and changing phone numbers [10, 11]. Another DAT known as 99 DOTs also has the potential to improve treatment adherence as the PWTB is required to call a hidden phone number within the blister pack so as to indicate when a dose was taken [10]. However, 99 DOTS is limited in its ability to accurately report if a dose was taken since the PWTB may dial the number without actually taking any medication [10, 12]. This challenge has been overcome by DATs methods like Video DOT (VOT) which allow HCWs to watch the PWTB taking treatment over video-conferencing hence it is considered a more accurate method to ensure that PWTB adhere to treatment [13]. In addition, VOT can be a source of support to those who lack family and friends to support them during their TB illness [14]. However, VOT requires a smartphone, data plan and sufficient bandwidth making it a less affordable method in low and middle income countries such as SA [13].

For peer review only - http://bmjopen.bmj.com/site/about/guidelines.xhtml

Most DATs have focused on monitoring adherence and few studies have used DATs to differentiate between those patients who are struggling and need individual support, and those who are doing well. Given this gap in knowledge, we evaluated the feasibility of using the Wisepill evriMED DAT to monitor TB treatment adherence while using a differentiated care approach (DCA) with a stepwise increase in adherence support for PWTB in South Africa.

METHODS

Study design and study setting

This qualitative study was embedded within a cluster-randomized trial (CRT) that took place in six clinics in each of three provinces of South Africa: Gauteng (Ekurhuleni district); Western Cape (Klipfontein and Mitchell's Plain districts); and Kwa-Zulu Natal (eThekwini district), the details of which have been published elsewhere [15]. In the intervention arm of the CRT, PWTB received medication monitors with reminders triggering differentiated care approach (DCA) in response to adherence data uploads, carried out from a central database [15]. The DCA was implemented in a progressive manner depending on the number of doses a participant missed [15]. If one dose was missed, then a short message service (SMS) reminder was sent to the participant [15]. If a second or third dose was missed, then study staff would make a telephone call to the participant and once the fourth dose was missed then a home visit was conducted during which motivational counselling took place [15]. We describe the *feasibility* of implementing Wisepill evriMED device and differentiated care from a stakeholder perspective.

Site selection

There were a total of nine intervention clinics, three in each province. Clinics were selected based on location, HIV prevalence and numbers of patients starting TB treatment per month [15].

For peer review only - http://bmjopen.bmj.com/site/about/guidelines.xhtml

Study population

The study population consisted of seven purposively selected stakeholders from the Department of Health (DOH): one national, one provincial and five district-level representatives, who worked closely with the facilities using the electronic device (Wisepill evriMED DAT). From each intervention facility, we interviewed one facility staff (government employee) and one study staff member. All study staff worked on the project for at least three months. Facility staff-initiated patients on TB treatment and monitored patients for their scheduled monthly follow-up visits while study staff offered PWTB the device, followed-up on those who had missed doses and provided motivational and adherence counselling.

Patient and public involvement

Patients or the public were not involved in the design, or conduct, or reporting, or dissemination plans of our research.

Conceptual framework and themes explored

Using the feasibility framework suggested by Bowen et al [16], we developed an in depth interview (IDI) guide (Appendix I) that covered the following topics; (i) the relative ease of implementation and operation of the technology within existing health systems, technology infrastructure and supply chain; (ii) system level challenges of delivering, sustaining and integrating the intervention into the existing TB programme. We used the framework on the 'Integration and sustainability of interventions into health systems' [17] to organize the data.

Data collection

We conducted a total of 25 in-depth interviews. The sample was representative of providers involved in the intervention while also considering saturation where themes usually start to converge after 15 interviews. The IDI guide (Appendix I) was piloted with one stakeholder,

For peer review only - http://bmjopen.bmj.com/site/about/guidelines.xhtml

two facility and one study staff between January to February 2020. The probing questions were adapted after the pilot. These interviews were conducted face-to-face, and the data was included in the analysis. Data collection continued between June 2020 to January 2021 with each interview lasting between 45 – 90 minutes. The interviews were all conducted in the providers preferred language by a female PhD student (RM) and a female study coordinator who was a PhD student with qualitative experience (VM). Both researchers established a prior relationship with the providers enabling them to understand the reasons for the study. The interviews were digitally recorded with the providers consent and transcribed verbatim by trained research assistants (Masters students). The transcripts were not returned to the providers for comment or correction. Due to the impact of COVID-19 and the national lockdown that took place, these interviews were conducted virtually over Microsoft Teams. No one else was present in the interviews besides the providers and the researchers and field notes were made during the interviews. Saturation was assessed during data collection through asking the same question in different ways and reviewing a sample of the recordings until no new information was obtainable. No repeat interviews were carried out.

Data analysis

Thematic analysis was used with deductive and inductive approaches [18, 19]. At least 10% of the transcripts were coded inductively by two independent researchers so as to reduce bias and improve reliability [18]. A codebook of emerging themes was developed, guided by the framework on the ‘Integration and sustainability of interventions into health systems’ [17],with discrepancies being resolved through discussion. Where there was no inter-rater agreement, the theme was dropped [18]. The final codebook was used to code the remaining transcripts and any new codes that emerged were also included in this codebook. MAXQDA qualitative software was used for the coding process. We highlight the major themes (overarching theme) and sub-themes (specific themes) that emerged and use

For peer review only

supportive direct quotations from providers. Providers did not provide feedback on the findings.

RESULTS

We conducted 25 interviews in total with 18 staff (9 facility staff and 9 study staff) and 7 stakeholders (1 national,1 provincial and 5 at district-level) across 3 provinces. Majority of the providers (23 out of 25) were female. None of the approached providers declined to participate. Major themes included (1) Providers were supportive of the intervention, (2) Intervention challenges within the adoption system and broad context, and (3) Ensuring intervention sustainability through constant training of staff and education of PWTB

Major Theme 1: Providers were supportive of the intervention

There was buy-in from staff who felt involved and were supportive of the intervention. Stakeholders were also very supportive of the intervention despite their limited involvement.

> *"… to me it was a very good idea and it was working very well … it improved our cure rate because we had a lot of patients who were defaulting and who and to be referred to the hospital because they developed multi, MDR. So since we had …this intervention, at least we had less patients who developed multi-drug resistance."*
> *- FFS_019_7*

> *"…I think…it's magnificent. I think it's… a tool box, it's really a way for us to see what's really happening at the point of … TB treatment, where the patient take… the medication when they open the box. So I think it's really innovation that can be used. ... I think it also is a reminder for the patient. You know? That … "I need to take my medication". And it's a way to …to ensure the quality of the programme, that patients are adhering to the ... medication…I think it is a critical intervention." – FDS_WC_008 (District level)*

For peer review only - http://bmjopen.bmj.com/site/about/guidelines.xhtml

Both facility and study staff found the device easy to learn and facility staff who had received a briefing on the study were eager to receive the complete training to support the study staff.

> *"Because sometimes she (referring to study staff) [might not be here and it happens that she's not here, either she is sick or she's gone on holiday for December. She says: "…we can show you and then you do this things when we are not here". I said: "… it will be easy… As long as you show us, we can do that device".*
> *- FFS_762_003*

The staff described the activities of the intervention such as issuing devices, phone calls and making home visits as being well integrated into the TB programme. Providers had strong support for the device for two main reasons that we classified as sub-themes in our analysis:

Sub-theme 1: Device as a useful reminder and early notification tool

Most providers found the alarm that was fitted on the device to be a useful reminder to patients to take their treatment on time and to attend their clinic visits. The device also alerted providers to PWTB who were not using the device and not taking treatment.

> *"I feel like it assists with the real time monitoring because now if you check on the system, you would be able to check instead of waiting like someone waiting for their appointment that is months after, you can actually check now on the system that this person there's no activity, let me call and you would actually find that the person has died or maybe the person has- is in hospital is admitted.."- FSS_762_006*

> *"So, it will help us because …sometimes we notice the other patient…didn't take their medication and start to recall all of them and then the other just default … With the*

For peer review only - http://bmjopen.bmj.com/site/about/guidelines.xhtml

box, someone is getting … a notification that this person is not taking her treatment...” - FFS_524_006

However, some staff distrusted that opening of the device meant that PWTB took their pills.

“…some patients do that when they open the pill box and … show an adherence of 100%, but still they… have not been taking their medications. So we would try to motivate them, counsel them, explain the disadvantages of not of them not taking the medication.” - FSS_519_001

“I think because you know patients always find a way to find loopholes within the health system, so at some point they will understand that you’re recording the opening and the closing of the box not necessarily them actively taking the medications. So they can easily open and close the box without really taking the medication.” - FDS_GP_008 (District level)

Sub-theme 2: Differentiated Care Approach allows for patient-centred care

Staff mentioned that they found DCA to be a positive and unique aspect of our intervention.

“So, you are no longer administering intervention for one. You sort of trying to be specific …to a person and offer them care in their in their specific sort of situations. So, I think that is a positive thing because you don’t assume everyone is the same. You don’t assume everyone’s situation is the same. You understand that you are dealing with … individuals. That’s … what I think is positive about this differentiated model of care.” - MSS_732_005

“… yah, I think I think the differentiated model of care … is a very positive thing, that it works to an extent because it’s not a blanket approach. You don’t, you don’t think

For peer review only - http://bmjopen.bmj.com/site/about/guidelines.xhtml

of every patient as the same…you sort of attend to each person in their context and … try to understand what they are going through." - MSS_732_005

The staff also felt that the counselling they provided as part of the DCA helped them to understand their patients' reasons for not taking their treatment thus allowing them to manage them better as they supported them on their treatment journey.

"This whole approach I feel like it's a great initiative because it assists in managing the patient - like fully. Like if I can say wholly, not just managing the patient, taking the medication. You could also like… with the counselling you actually find that the problem is that the participant is facing beyond treatment intake."- FSS_762_006

Phone calls to PWTB allowed for clarification on any issues that may not have been clear to the patient while at the clinic.

"…he didn't understand but when I called him and I was speaking to him over the phone to understand how they're taking the treatment, I find that … yeah there was mistake … one of the best things about this intervention because some of them are able to even call me up, I don't always now have to call patients."

- MSS_732_005

Home visits allowed the staff to have better interaction with their patients and to build trust with them making them feel like someone cares about them.

"It's not bad. It's nice to do home visits. It's whereby … you contact with the patients, know each other … where the patient has got the problem, it's with the … problem with the box or she wanted to ask me something that she forget to ask at the clinic."

- FSS_522_002

For peer review only - http://bmjopen.bmj.com/site/about/guidelines.xhtml

For peer review only

"… I think the participant at least get that thing when you do home visits, or you phone them when they are not taking the treatment they feel as if they are cared of, someone is care is cared for me when I am not taking the treatment because I would get a call, or I will get a home visit''- FSS_524_003

Some providers described DCA as more of a journey of supporting the patients and helping build trust between the staff and the PWTB.

"… that is really good part of the intervention. I think it … continue to build that trust…And that support for the patients … so I- I think that was impressive."
- FDS_WC_008 (District level)

"I thought it was only about the box… it's more than them just using the box. It's their treatment journey and being supported to adhere to the treatment and get healed from the TB." - FSS_762_006

Major Theme 2: Intervention challenges within the adoption system and broad context of South Africa

Some staff felt that the trust they built with the PWTB could be threatened by human resource shortages and a dedicated cadre was needed to ensure successful implementation.

"However, when we find at once the research study stop and we over onto implementation, … the constraints is always HR resources and at the operational level, if that initial education isn't framed correctly. There could be a misunderstanding about the purpose of the box." - FDS_WCR_008 (District level)

For peer review only - http://bmjopen.bmj.com/site/about/guidelines.xhtml

Challenges that were encountered with the device or the network resulted in delays in the system updating that the box was opened which staff felt created trust issues between them and the participant.

"Now it's creating a trust issue between you and the patient, because you phoning regarding treatment that was not taken because … Wisepill says treatment was not taken, while the patient on the other side did take the medication."- FSS_516_002

Heath care workers reported that some patients received incorrect SMS's due to delays in the system.

'' Especially when it has been a weekend and then they will not … send out the data of… the weekend like … if there were any missed or any … intakes. Sometimes they will show that they missed the weekend and they did not take the medication, but in three days down the line… the correct information will show. That's where we will see that there were no missed doses.''- FSS_519_001

Staff also felt that that the device would not work for some group of patients such as those who abuse substances.

"The patient that are on substance abuse, yoh! You know, when they come here, they are ok. At a later stage, once they've started treatment, you realise that this patient is using substances…With those patients, they the device- ya. It's not good. Won't- won't work. Won't- won't help." - FFS_762_003

"He took the evriMED box device and the TB meds. That was the last time we saw him on the clinic. He run away from home apparently. He was on drugs. But then…he is back. He's here in the TB room. He's got TB again. So I was asking him

For peer review only

the other day: "are you still interested in joining this study?". And he was like: "you know what I did with the first box? I sold it". - FSS_521_001

Sub-theme 1: Provider perceptions of stigma related to use of the evriMED device and DCA

Staff perceived that some of the features of the box such as the alarm and the size of the box may have been a concern to some PWTB in trying to conceal their TB status so they would opt to leave their devices behind when travelling on holiday or going to work for fear of disclosure of their TB status.

"The thing that is common, especially in December … an example is person supposed to take it at 8, they take it at 8 but they not taking it at 8 from the box... I think they went for holidays … This person is taking treatment and the putting it… in a purse. Whereby you'll take in the morning without this alarm ringing.''- MSS_019_002

"So some will come to us and say: "what if the pill box doesn't make so much noise? It will be easier for me to carry around- or if it was a bit … smaller, then it will be easier for me to carry it. But now it's big, I don't want … people seeing me like with this pill box in the taxi or at work."- FSS_519_001

"I haven't disclosed to my partner that I'm on TB medication and imagine if I had to carry this box and the box would be ringing in the morning and it would cause unnecessary fights." - FSS_762_006

Providers perceived that stigma was more of an issue outside the PWTB household as often they would not disclose their TB status to members outside their homes.

For peer review only - http://bmjopen.bmj.com/site/about/guidelines.xhtml

"So, I noticed that it's comfortable for them to use this box when they are at home with the people that actually know what is going on with them but when they are going to other people that are not aware that they are sick or anything, it seems as if they don't want to carry the boxes." - FSS_762_006

Staff perceived that stigma related to DCA existed in the community and some of them witnessed the PWTB fear of being stigmatized when conducting home visits. PWTB would often ask staff not to wear their uniforms when they visit their homes.

"Uhm they hate it when we wear our TB MATE t-shirts, because they are saying: "due to stigma". When we go visit their homes, we must not wear something that will be written 'TB' or 'HIV'. So we wear our normal clothes..."- FSS_521_001

"Then I took off my jacket, and then the participant saw that TB MATE on my t-shirt. And then the patient told me that I must wear my jacket because what if someone walks in and sees the TB MATE on my t-shirt. So I wasn't aware that the participant didn't disclose... She told me that she's got fear that- because she will be judged because she was drinking alcohol a lot. But now she's got TB. They will say she must go and stay in the back room."- FSS_521_001

Staff perceived that some stigma could have been internalised and not actually experienced by PWTB.

"It's their thoughts because the person would feel like it did not want to be seen carrying the box. Because people will think- so he sees all their thoughts. It's not something that really happened."- FFS_732_001

For peer review only - http://bmjopen.bmj.com/site/about/guidelines.xhtml

"But in the clinic, there wasn't really anything that contributed to the stigma but maybe with now giving the pill box… some of the patients would say that maybe it's gonna show. Maybe with their ARV's they could take it in private. Now we have them having this pill box that is gonna ring even, that is gonna beep and remind them to take treatment..." - FSS_732_005

Major Theme 3: Ensuring intervention sustainability through constant training of staff and education of PWTB

Education on the intervention emerged as one of the major themes that stakeholders and staff placed emphasis on as a means of managing their patients in a holistic way leading to successful treatment outcomes. Education and training should emphasize the importance of adhering to treatment and should be strengthened at various levels – with the PWTB themselves, those who would be involved in implementation such as community health workers (CHWs) and also to the community at large.

''My experience was that as the community we need to need to educate the community the importance of complying to medication…we need to educate and educate and educate.''- FFS_019_007

"But I definitely think it's… strengthening that interface between the patient and the staff... it's an important interface. And- and of course that too- the 'how' of- of the contact, if it is about 'why you didn't- why you aren't taking your treatment' rather than saying 'how are you doing'- you know- 'are you ok', uhm 'how can I help you'. When we start off with those different conversations, it can lead to different outcomes." - FDS_WC_008 (District level)

The staff felt that constant education and reinforcement would ultimately lead to behaviour change and treatment adherence.

For peer review only - http://bmjopen.bmj.com/site/about/guidelines.xhtml

“I think the intervention even though we are the one offering the intervention but we sort of gave patients the power to understand the… reasons why they are taking treatment, why is it important for them to…adhere to the treatment…we were actually able to make patients to take their treatment journey into their own hands … it’s almost like we were empowering patients…”- MSS_732_005

DISCUSSION

Using the Wisepill evriMED device to support adherence to TB treatment appears to be a feasible option in South Africa. Providers were supportive of the evriMED device and the differentiated care approach, as they felt it had a positive impact on the TB patient’s programme. Stakeholders were supportive despite their involvement being limited to approval of the study and providing oversight of the TB programme.

This study has shown that DATs is an innovative approach that might improve the management of PWTB by allowing individual differentiated support. Early notification of missed doses from the device allowed staff to intervene through SMS messaging and phone calls, thus reducing the need for a home visit. This freed up time for staff to focus on other duties. These findings are similar to other studies where differentiated care models used in HIV treatment delivery have addressed challenges such as overcrowded facilities, overburdened staff and long waiting times in countries like Uganda, Swaziland, Mozambique and South Africa [7, 20-22].

Staff noted that the differentiated care allowed a journey of educating and supporting PWTB. Through adherence and motivational counselling, staff empowered patients to understand the importance of adhering to treatment and to empower them to take their treatment journey into their own hands. For scale-up, the real time monitoring feature of the device is important

For peer review only - http://bmjopen.bmj.com/site/about/guidelines.xhtml

so that the correct individuals are promptly identified for further support before they become lost to follow-up. This also allows for the efficient utilisation of resources to those in most need, as seen in various studies [6, 23, 24]. More attention should also be given to relationship training as the sustainability of the intervention depends on a sound provider-patient relationship. Correct framing of the intervention as a support tool would also help ensure that the intervention is well perceived once scaled-up. Training needs to be constant and not once-off to reinforce their understanding of the intervention. In a routine TB program setting, training of healthcare providers could be conducted by sub-district co-ordinators since they visit facilities on a regular basis for monitoring purposes.

Some challenges with the technology were cited such as alarm malfunction, incorrectly sent SMS's and short battery lifespan. These challenges created distrust from PWTB, and the device would therefore need to be of the highest quality to ensure sustainability of the intervention. A systematic review of DAT for the management of tuberculosis therapy found similar feasibility challenges remain in low- and middle-income settings [10].Once the intervention is scaled up, the shortage of human resources (HR) could serve as a barrier to ensuring that providers receive the correct information. For successful scale-up, some staff recommended that a dedicated cadre should be in place to follow-up PWTB to ensure that they understand the importance of taking treatment. However, since the support system had reduced the need for home visits and follow up calls, the current TB cadres, clerical staff and community-based teams were also seen as being sufficient. Despite any challenges experienced within the adoption system and broad context, the staff were all very supportive of the intervention and even recommended that it should be used for all TB and chronic patients as well as in other facilities.

Fear of stigma and the disclosure of one's TB status has the potential to act as a barrier to scale-up and sustainability of the intervention, if not well addressed. Stigma and fear of disclosure resulting in lower acceptance of DAT technologies amongst multi-drug resistant

For peer review only - http://bmjopen.bmj.com/site/about/guidelines.xhtml

TB patients have also been reported in India [25], with improvements recommended on the design. Disease-related stigma may be more difficult to address, and screening should be used to identify upfront those patients whom stigma and fear of disclosure may limit the use of the MERM [25].

A limitation of the study was that interviews had to be conducted virtually on Microsoft teams, due to the Covid-19 pandemic, and to allow for effective social distancing for both the researchers and the study providers. This new mode of conducting interviews had setbacks such as network and connectivity challenges which interrupted the flow of some of the interviews. The interviewers mitigated this challenge through re-iterating what the participant had said to ensure that the correct message had been captured. Through the consenting process, we were able to ensure confidentiality and that the participant was relaxed. A second limitation is that some of the interviews were conducted by a member of the study management team, although this was limited to senior stakeholders who were unlikely to feel reluctant to freely express their views and to withhold some of their opinions. Patients were not included in this study which is a limitation since their views would have added a broader understanding on feasibility. We conducted the study in three provinces with different health service characteristics, TB epidemiology and population characteristics which allowed us to understand feasibility of implementation from a broader perspective. Another strength was that the study was done in a routine setting with limited resources and without the use of incentives to providers to increase uptake.

CONCLUSION

Digital adherence technology (DAT) such as medication monitors have a huge potential to improve tuberculosis treatment adherence. However, to ensure successful scale-up and sustainability of the intervention, the differentiated care approach should be used as a platform to constantly educate PWTB and the community at large on TB and the importance

For peer review only - http://bmjopen.bmj.com/site/about/guidelines.xhtml

of adhering to treatment since technology on its own will not solve treatment adherence issues especially those related to stigma and lack of support. Sound communication between PWTB and providers serves as a key tool to improving treatment outcomes thus more attention should be given to relationship training to ensure that the provider-patient relationships provide the necessary support to those who need it most.

ABBREVIATIONS

COVID	Corona Virus Disease
DCA	Differentiated Care Approach
DOH	Department of Health
DS-TB	Drug-sensitive TB
DATs	Digital Adherence Technologies
DOTs	Directly Observed Treatment Short Course
ICFs	Informed Consent Forms
IDI	In-depth Interview
MDR-TB	Multidrug-resistant TB
PWTB	People With TB
SA	South Africa
SMS	Short Message Service
TB	Tuberculosis
TB MATE	TB Monitoring Adherence to Treatment Endpoints

ACKNOWLEDGEMENTS

We would like to thank the following: Ekurhuleni, City of Cape Town, eThekwini districts and the Ekurhuleni Health District Research Committee (EHDRC) for allowing us to conduct the study in their districts. The study coordinators from Gauteng, Western Cape and KwaZulu-Natal in South Africa for their assistance with data collection namely Israel Rabothata, Vuyelwa Mehlomakulu (VM), Vumile Gumede and Bongani Zondi and their field-based teams of research assistants and interns; all the transcribers who dedicated time to capture the conversations verbatim; all the providers for taking part in this study

CONTRIBUTORSHIP STATEMENT

RM, NM, LJ, MTM, KLF, SC and CMCM conceptualized the study. RM and CMCM analysed the data. RM, NM, CO, SC and CMCM interpreted the data. RM wrote the original draft and revised subsequent versions of the manuscript. SC and CMCM provided guidance on earlier versions of the manuscript. NM, CO, LJ, PN, MTM, KV, KLF, SC and CMCM reviewed and edited previous versions. RM, SC and CMCM are responsible for the overall content of the manuscript. The authors read and approved the final manuscript.

For peer review only - http://bmjopen.bmj.com/site/about/guidelines.xhtml

COMPETING INTERESTS

The authors declare that they have no competing interests.

FUNDING STATEMENT

The study is funded by (1) Bill & Melinda Gates Foundation (OPP1205388), (2) TB REACH Wave 6 project of Stop TB Partnership (STBP/TBREACH/GSA/W6-34), and (3) South African Medical Research Council through the Strategic Health Innovation Partnerships. Funders did not play a part in the design of the study or the decision to submit the manuscript for publication.

DATA SHARING STATEMENT

No data are available.

ETHICS APPROVAL AND PARTICIPANT CONSENT

The parent study received ethics approval from the three district and provincial ethics committees where the trial sites were located. This qualitative study was approved by the Human Research Ethics Committee (HREC) of the University of Witwatersrand (Ref 180705) and the University of Cape Town (Ref 452/2018). We obtained written informed consent for study participation and permission for digital recording from all providers. Providers were not reimbursed.

REFERENCES

1. WHO. Global Tuberculosis Report 2021. Geneva: World Health Organization 2021 [Available from: https://worldhealthorg.shinyapps.io/tb_profiles/?_inputs_&entity_type=%22country%22&lan=%22EN%22&iso2=%22ZA%22.

2. WHO. Global Tuberculosis Report 2018. Geneva: World Health Organization.

3. Cramm JM, Finkenflugel HJ, Moller V, Nieboer AP. TB treatment initiation and adherence in a South African community influenced more by perceptions than by knowledge of tuberculosis. BMC Public Health. 2010;10:72.

4. Knight M, van Zyl RL, Sanne I, Bassett J, van Rie A. Impact of combination antiretroviral therapy initiation on adherence to antituberculosis treatment. South Afr J HIV Med. 2015;16(1):346.

5. Ntshanga SP, Rustomjee R, Mabaso ML. Evaluation of directly observed therapy for tuberculosis in KwaZulu-Natal, South Africa. Trans R Soc Trop Med Hyg. 2009;103(6):571-4.

6. Serapelwane MG, Davhana-Maselesele M, Masilo GM. Experiences of patients having tuberculosis (TB) regarding the use of Directly Observed Treatment Short-Course (DOTS) in the North West Province, South Africa. Curationis. 2016;39(1):e1-e9.

7. Liu X, Lewis JJ, Zhang H, Lu W, Zhang S, Zheng G, et al. Effectiveness of Electronic Reminders to Improve Medication Adherence in Tuberculosis Patients: A Cluster-Randomised Trial. PLoS Med. 2015;12(9):e1001876.

8. Bionghi N, Daftary A, Maharaj B, Msibi Z, Amico KR, Friedland G, et al. Pilot evaluation of a second-generation electronic pill box for adherence to Bedaquiline and antiretroviral therapy in drug-resistant TB/HIV co-infected patients in KwaZulu-Natal, South Africa. BMC Infect Dis. 2018;18(1):171.

For peer review only - http://bmjopen.bmj.com/site/about/guidelines.xhtml

9. Hüsler J. Evaluation of the On Cue Compliance Service Pilot : Testing the use of SMS reminders in the treatment of Tuberculosis in Cape Town, South Africa. 2005.
10. Subbaraman R, de Mondesert L, Musiimenta A, Pai M, Mayer KH, Thomas BE, et al. Digital adherence technologies for the management of tuberculosis therapy: mapping the landscape and research priorities. BMJ Glob Health. 2018;3(5):e001018.
11. Hermans SM, Elbireer S, Tibakabikoba H, Hoefman BJ, Manabe YC. Text messaging to decrease tuberculosis treatment attrition in TB-HIV coinfection in Uganda. Patient Prefer Adherence. 2017;11:1479-87.
12. Cross A, Gupta N, Liu B, Nair V, Kumar A, Kuttan R, et al. 99DOTS: A Low-Cost Approach to Monitoring and Improving Medication Adherence. Tenth International Conference on Information and Communication Technologies and Development (ICTD '19). 2019.
13. Story A, Aldridge RW, Smith CM, Garber E, Hall J, Ferenando G, et al. Smartphone-enabled video-observed versus directly observed treatment for tuberculosis: a multicentre, analyst-blinded, randomised, controlled superiority trial. Lancet. 2019;393(10177):1216-24.
14. Fraser H, Keshavjee S. Video-observed therapy for tuberculosis: strengthening care. Lancet. 2019;393(10177):1180-1.
15. Maraba N, Orrell C, Chetty-Makkan CM, Velen K, Mukora R, Page-Shipp L, et al. Evaluation of adherence monitoring system using evriMED with a differentiated response compared to standard of care among drug-sensitive TB patients in three provinces in South Africa: a protocol for a cluster randomised control trial. Trials. 2021;22(1):389.
16. Bowen DJ, Kreuter M, Spring B, Cofta-Woerpel L, Linnan L, Weiner D, et al. How we design feasibility studies. Am J Prev Med. 2009;36(5):452-7.
17. Atun R, de Jongh T, Secci F, Ohiri K, Adeyi O. Integration of targeted health interventions into health systems: a conceptual framework for analysis. Health Policy Plan. 2010;25(2):104-11.
18. Pope C, Ziebland S, Mays N. Qualitative research in health care. Analysing qualitative data. BMJ. 2000;320(7227):114-6.
19. Charmaz K. Constructing Grounded Theory: A practical guide through qualitative analysis Nurse Res. 2006;13(4):84.
20. Wilkinson L, Harley B, Sharp J, Solomon S, Jacobs S, Cragg C, et al. Expansion of the Adherence Club model for stable antiretroviral therapy patients in the Cape Metro, South Africa 2011-2015. Trop Med Int Health. 2016;21(6):743-9.
21. Decroo T, Telfer B, Biot M, Maikere J, Dezembro S, Cumba LI, et al. Distribution of antiretroviral treatment through self-forming groups of patients in Tete Province, Mozambique. J Acquir Immune Defic Syndr. 2011;56(2):e39-44.
22. Okoboi S, Ding E, Persuad S, Wangisi J, Birungi J, Shurgold S, et al. Community-based ART distribution system can effectively facilitate long-term program retention and low-rates of death and virologic failure in rural Uganda. AIDS Res Ther. 2015;12:37.
23. Kurz SG, Furin JJ, Bark CM. Drug-Resistant Tuberculosis: Challenges and Progress. Infect Dis Clin North Am. 2016;30(2):509-22.
24. Subbaraman R, Nathavitharana RR, Mayer KH, Satyanarayana S, Chadha VK, Arinaminpathy N, et al. Constructing care cascades for active tuberculosis: A strategy for program monitoring and identifying gaps in quality of care. PLoS Med. 2019;16(2):e1002754.
25. Thomas BE, Kumar JV, Periyasamy M, Khandewale AS, Hephzibah Mercy J, Raj EM, et al. Acceptability of the Medication Event Reminder Monitor for Promoting Adherence to Multidrug-Resistant Tuberculosis Therapy in Two Indian Cities: Qualitative Study of Patients and Health Care Providers. J Med Internet Res. 2021;23(6):e23294.

For peer review only - http://bmjopen.bmj.com/site/about/guidelines.xhtml

APPENDIX I

STUDY NUMBER: -☐☐☐-☐☐☐☐☐-☐ **AUR2-1-244**

INTERVIEW GUIDE:

DEPARTMENT OF HEALTH KEY STAKEHOLDERS/ FACILITY-BASED STAFF/STUDY STAFF

TB MATE

Introduction

Staff to say out loud before starting interview

- Thank you for agreeing to take part in our research
- We are interested in knowing about your perceptions, thoughts and attitude toward the differentiated model of care, especially with regard to implementation of the medication device. There are no right or wrong answers.
- You do not have to answer any questions if you don't want to. Just say pass and I will move to the next question.
- Your answers to these questions may help us improve TB treatment services.
- All of your answers will be kept confidential. Confidential means we will not tell anyone your answers outside the research group.

For peer review only

For peer review only - http://bmjopen.bmj.com/site/about/guidelines.xhtml

APPENDIX I

STUDY NUMBER: -□□□-□□□□□-□ **AUR2-1-244**

INTERVIEW GUIDE:

DEPARTMENT OF HEALTH KEY STAKEHOLDERS/ FACILITY-BASED STAFF/STUDY STAFF

Introduction and Ground Rules

1. Obtain written informed consent first, before any data are collected.

2. Interviewer to introduce self. Thank you for taking the time to meet with us today. Our names are [*insert names*]___________and we would like to talk to you about the feasibility, acceptability and the quality to which the differentiated care model was implemented. We are doing this project to understand your role, thoughts, attitude and perceptions of the differentiated model of care and particularly to see whether the medical device technology could be integrated and sustained within the TB programme. We want you to be as open and honest when answering. There are no right or wrong answers in this discussion. Please feel free to tell us what you think.

    - Interviewer to explain the ground rules and terms of confidentiality for the interview.
    - The participant does not have to answer any question they do not want to.
    - The information you share will be handled in confidence. (in secret)
    - When we report back on the information collected in this discussion, your comments will not be able to be linked to you specifically.
    - We ask that you also agree not to share anything discussed in this room with others.

3. The interview should take about 1 hour

4. Interviewer to inform the interviewee that the in-depth interview will be digitally recorded to make sure that all themes are captured. Turn the audio-recorder on and ask for verbal permission again to digitally record the session. While the audio-recorder is running verbal consent (this is a double check against the written consent) must be captured. We will be recording the session because we don't want to miss any of your comments. Although one of us may take some notes while we talk, we can't write fast enough to get everything down on paper. As we are recording, please try to speak loudly so that we don't miss your comments.

Themes to be explored

I. Feasibility of implementing the differentiated model of care (motivators and barriers)

For peer review only - http://bmjopen.bmj.com/site/about/guidelines.xhtml

APPENDIX I

STUDY NUMBER: -□□□-□□□□□-□ AUR2-1-244

INTERVIEW GUIDE:

DEPARTMENT OF HEALTH KEY STAKEHOLDERS/ FACILITY-BASED STAFF/STUDY STAFF

II. System level challenges of delivering, sustaining and integrating the intervention into the existing TB programme

For peer review only

Time started (HHMM):

Questions

1. **General**

- What is the title of your current position?
- How long have you held this position?
- How are TB services delivered at your level with regard to the intervention? (i.e. District, Provincial, National)
- Please describe your role with the differentiated model of care intervention

2. **Feasibility of implementing the differentiated model of care (motivators and barriers)**

- *Please describe the cadre of staff that were involved with delivering the differentiated model of care?*
- *What are your thoughts on features of the Evrimed device?*
- *Can you describe your experience of delivering the differentiated model of care?*
- *Can you describe the training and resources that staff received prior to or during delivery of the differentiated model of care? What was your opinion of the training and resources received?*
- *Can you describe the benefits of the differentiated model of care and use of the medication device technology?*
- *How often did you follow the requirements for the differentiated model of care?*
- *Please describe the details of what happens on the phone calls and at the home visits.*
- *What tools do you use for the motivational counselling and for adherence counselling?*

For peer review only - http://bmjopen.bmj.com/site/about/guidelines.xhtml

APPENDIX I

STUDY NUMBER: -□□□-□□□□□-□ **AUR2-1-244**

INTERVIEW GUIDE:

DEPARTMENT OF HEALTH KEY STAKEHOLDERS/ FACILITY-BASED STAFF/STUDY STAFF

- *Can you describe the differences between motivational counselling and adherence counselling?*
- *Under what circumstances were not able to follow the requirements for the differentiated model of care?*
- *How can the differentiated model of care be sustained?*
- *Can you describe challenges of the differentiated model of care and use of the medication device technology?*
- *How can TB treatment be improved using this differentiated model of care and the medication device technology?*
- *What was your experience or suggestions regarding interactions with patients? (Giving instructions, or providing motivational counselling)*

3. System level challenges of delivering the intervention

- *How were patients that were part of the differentiated model of care treated differently from those receiving standard of care?*
- *Please elaborate on the positive changes of the differentiated model of care and use of the medication device technology. How do you think these positive changes could be sustained?*
- *Please elaborate on the negative changes of the differentiated model of care and use of the medication device technology. How do you think the negative changes could be addressed?*
- *Can you describe to us what systems are in place that could monitor the differentiated model of care and use of the medication device technology?*
- *Were there any activities that caused or contributed to patients experiencing stigma at the facility? Please elaborate*
- *What types of resources will facilities need to ensure sustainability of the differentiated model of care?*
- *Please describe to us what system level structures need to be improved in order to integrate the differentiated model of care and medication device technology into the existing TB programme system.*

4. Factors that influence the sustainability of the intervention

- *Please describe to us the expectations that you may have had about the differentiated model of care and use of the medication device technology?*

For peer review only - http://bmjopen.bmj.com/site/about/guidelines.xhtml

For peer review only

APPENDIX I

STUDY NUMBER: -□□□-□□□□□-□ **AUR2-1-244**

INTERVIEW GUIDE:

DEPARTMENT OF HEALTH KEY STAKEHOLDERS/ FACILITY-BASED STAFF/STUDY STAFF

- *Please describe your positive and negative experience of delivering or implementing the differentiated model of care.*
- *How different is the current intervention model compared to your initial expectations and how do you think this model could be more effective?*
- *For scale-up and sustainability who do you think should set up the Evrimed boxes for patients? What would be the advantages and disadvantages of this cadre of staff setting up the Evrimed boxes as part of their daily responsibilities.*
- *Can you describe any gaps which exist in the way the intervention is being delivered currently?*

Any other comments

Are there any final thoughts you have about the differentiated model of care and use of the medication device technology?

End of session

Now we have come to the end of our discussion. Thank you for your participation. If you have any questions about your study participation, please contact us. Thank you.

Time ended (HHMM):

For peer review only

For peer review only - http://bmjopen.bmj.com/site/about/guidelines.xhtml

COREQ (COnsolidated criteria for REporting Qualitative research) Checklist

A checklist of items that should be included in reports of qualitative research. You must report the page number in your manuscript where you consider each of the items listed in this checklist. If you have not included this information, either revise your manuscript accordingly before submitting or note N/A.

Topic	Item No.	Guide Questions/Description	Reported on Page No.
**Domain 1: Research team and reflexivity**			
Personal characteristics			
Interviewer/facilitator	1	Which author/s conducted the interview or focus group?	
Credentials	2	What were the researcher's credentials? E.g. PhD, MD	
Occupation	3	What was their occupation at the time of the study?	
Gender	4	Was the researcher male or female?	
Experience and training	5	What experience or training did the researcher have?	
Relationship with participants			
Relationship established	6	Was a relationship established prior to study commencement?	
Participant knowledge of the interviewer	7	What did the participants know about the researcher? e.g. personal goals, reasons for doing the research	
Interviewer characteristics	8	What characteristics were reported about the inter viewer/facilitator? e.g. Bias, assumptions, reasons and interests in the research topic	
**Domain 2: Study design**			
Theoretical framework			
Methodological orientation and Theory	9	What methodological orientation was stated to underpin the study? e.g. grounded theory, discourse analysis, ethnography, phenomenology, content analysis	
Participant selection			
Sampling	10	How were participants selected? e.g. purposive, convenience, consecutive, snowball	
Method of approach	11	How were participants approached? e.g. face-to-face, telephone, mail, email	
Sample size	12	How many participants were in the study?	
Non-participation	13	How many people refused to participate or dropped out? Reasons?	
Setting			
Setting of data collection	14	Where was the data collected? e.g. home, clinic, workplace	
Presence of non-participants	15	Was anyone else present besides the participants and researchers?	
Description of sample	16	What are the important characteristics of the sample? e.g. demographic data, date	
Data collection			
Interview guide	17	Were questions, prompts, guides provided by the authors? Was it pilot tested?	
Repeat interviews	18	Were repeat inter views carried out? If yes, how many?	
Audio/visual recording	19	Did the research use audio or visual recording to collect the data?	
Field notes	20	Were field notes made during and/or after the inter view or focus group?	
Duration	21	What was the duration of the inter views or focus group?	
Data saturation	22	Was data saturation discussed?	
Transcripts returned	23	Were transcripts returned to participants for comment and/or	

For peer review only

Topic	Item No.	Guide Questions/Description	Reported on Page No.
		correction?	
**Domain 3: analysis and findings**			
Data analysis			
Number of data coders	24	How many data coders coded the data?	
Description of the coding tree	25	Did authors provide a description of the coding tree?	
Derivation of themes	26	Were themes identified in advance or derived from the data?	
Software	27	What software, if applicable, was used to manage the data?	
Participant checking	28	Did participants provide feedback on the findings?	
Reporting			
Quotations presented	29	Were participant quotations presented to illustrate the themes/findings? Was each quotation identified? e.g. participant number	
Data and findings consistent	30	Was there consistency between the data presented and the findings?	
Clarity of major themes	31	Were major themes clearly presented in the findings?	
Clarity of minor themes	32	Is there a description of diverse cases or discussion of minor themes?	

Developed from: Tong A, Sainsbury P, Craig J. Consolidated criteria for reporting qualitative research (COREQ): a 32-item checklist for interviews and focus groups. *International Journal for Quality in Health Care*. 2007. Volume 19, Number 6: pp. 349 – 357

Once you have completed this checklist, please save a copy and upload it as part of your submission. DO NOT include this checklist as part of the main manuscript document. It must be uploaded as a separate file.

For peer review only
